# Nanoceria as Next-Generation Immunotherapeutics: Applications in Chronic Inflammation, Cancer, and Tissue Repair

**DOI:** 10.3390/jnt6040028

**Published:** 2025-10-04

**Authors:** Kay Hadrick, Panangattukara Prabhakaran Praveen Kumar, Taeho Kim

**Affiliations:** Department of Biomedical Engineering, Institute for Quantitative Health Science and Engineering, Michigan State University, East Lansing, MI 48824, USA

**Keywords:** nanoceria, reactive oxygen species, immune response, therapy, chronic inflammation, wound healing

## Abstract

The immune system is crucial in protecting against disease, but it can also contribute to chronic illnesses when it malfunctions, with different conditions involving either inflammation or immune suppression. Current treatments often fall short due to limited effectiveness and side effects. Nanomedicine, particularly cerium oxide nanoparticles (nanoceria), offers promising potential due to its unique therapeutic properties and role in modulating macrophages. Nanoceria (<5 nm) possess the catalytic ability to mimic natural enzymes such as superoxide dismutase, peroxidase, and catalase, enabling effective scavenging of reactive oxygen species (ROS), which play a central role in the pathogenesis of chronic inflammation and cancer. This review comprehensively summarizes the current advances in the application of nanoceria for inflammatory and anti-inflammatory therapy, including their modulatory effects on immune cell activation, cytokine production, and resolution of inflammatory responses. We discuss the mechanisms underlying their immunomodulatory actions in various disease contexts, such as rheumatoid arthritis, women’s health conditions (e.g., endometriosis), wound healing, and cancer. Additionally, the review highlights biocompatibility, therapeutic efficacy, adaptability in imaging (theranostics), and challenges in translating nanoceria-based therapies into clinical practice. The multifunctionality of nanoceria positions them as innovative candidates for next-generation immunotherapy aimed at efficiently controlling inflammation and promoting tissue repair.

## Graphical Abstract

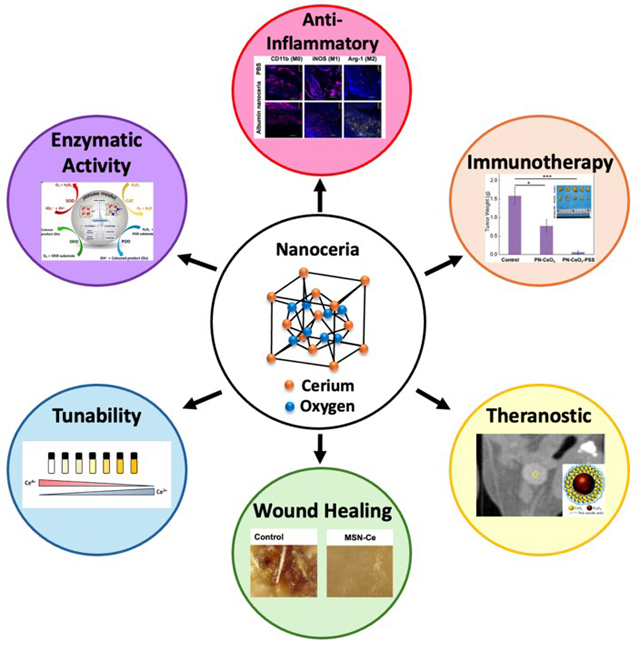

## 1. Introduction

Immunotherapy has emerged as a transformative modality in modern medicine, offering new avenues for treating a wide range of disease conditions, including cancer, autoimmune disorders, and chronic inflammation [1]. By modulating or enhancing the body’s own immune responses, immunotherapy can exhibit durable and targeted effects on disease. Despite its growing clinical relevance, current immunotherapies are often hindered by limitations such as off-target effects, immune-related adverse events, poor response rates in certain patient populations, and the immunosuppressive nature of some disease microenvironments [2,3]. These challenges underscore the urgent need for alternative innovative technologies that can improve the efficacy, specificity, and safety profile of immunotherapeutic interventions [4].

Nanotechnology has evolved as a powerful tool to address many of these shortcomings, offering precise delivery systems, controlled drug release, and the ability to alter the immune function and response at the cellular and molecular levels [5,6]. Among the diverse nanomaterials under investigation, cerium oxide nanoparticles, commonly referred to as nanoceria, have attracted significant attention due to their unique physicochemical and biological properties [7,8]. Unlike traditional nanocarriers, nanoceria possesses intrinsic catalytic activity resulting from the reversible switch between Ce^3+^ and Ce^4+^ oxidation states on its surface. This redox cycling endows nanoceria with robust reactive oxygen species (ROS) scavenging and anti-inflammatory capabilities, positioning it as an ideal candidate for regulating immune responses in disease settings characterized by oxidative stress and immune dysregulation.

Recent advances have expanded the scope of nanoceria applications beyond its antioxidant role, revealing its potential as an active immunomodulator [9,10]. Functionalization of nanoceria with targeting ligands, therapeutic agents, or immune checkpoint modulators has further enabled site-specific action and improved bioavailability. Additionally, nanoceria’s ability to modulate the polarization of macrophages, regulate cytokine release, and influence antigen-presenting cells suggests broader applications in shaping both innate and adaptive immune responses [11,12]. These properties open new possibilities for integrating nanoceria into combination immunotherapies, enhancing their efficacy while minimizing systemic toxicity.

This review aims to provide a comprehensive overview of the advancements in the development and application of nanoceria in the context of immunotherapy. We begin by discussing the synthetic routes and fundamental properties of nanoceria that underlie its biomedical utility, including redox behavior, surface chemistry, and biocompatibility. We then examine its immunomodulatory effects, including interactions with immune cells and signaling pathways. Key preclinical and emerging clinical studies are highlighted to underscore current progress, followed by a discussion of major challenges, such as long-term toxicity, biodistribution, and regulatory considerations. Finally, we offer perspectives on future directions and opportunities for integrating nanoceria into next-generation immunotherapeutic platforms.

## 2. Nanoceria as Potential Nanomedicine

Cerium, which holds the atomic number 58 on the periodic table, is a lanthanide and a rare earth metal characterized by its light gray, soft, and highly reactive nature in its pure solid form [13]. It commonly forms two types of oxides: CeO_2_, where cerium is in the +4 oxidation state, and Ce_2_O_3,_ with cerium in the +3 state. These cerium oxides have distinct appearances and properties; CeO_2_ appears almost white or pale yellow, while Ce_2_O_3_ exhibits a richer yellow-gold [14]. Among these, CeO_2_ is more prevalent and interestingly contains some Ce^3+^ ions, which enhance its catalytic properties [15]. Nanoscale cerium oxide (nanoceria) has gained attention for its medical applications due to its unique ability to switch between Ce^3+^ and Ce^4+^ on its surface, conferring enzyme-like antioxidant activity and the capacity to neutralize reactive oxygen species (ROS), thereby offering long-lasting anti-inflammatory effects [16]. In nanoparticle form, cerium oxide surfaces display a mixture of Ce^3+^ and Ce^4+^ ions. As the particle size decreases, the number of surface oxygen vacancies and Ce^3+^ ions increases; this occurs because, to compensate for surface oxygen loss, two cerium ions transition from Ce^4+^ to Ce^3+^ [17,18]. The oxygen vacancies, also known as “catalytic hot spots”, exhibit a size-dependent increase, with smaller nanoparticles tending to have a higher Ce^3+^ content [8,19].

**Figure 1.**
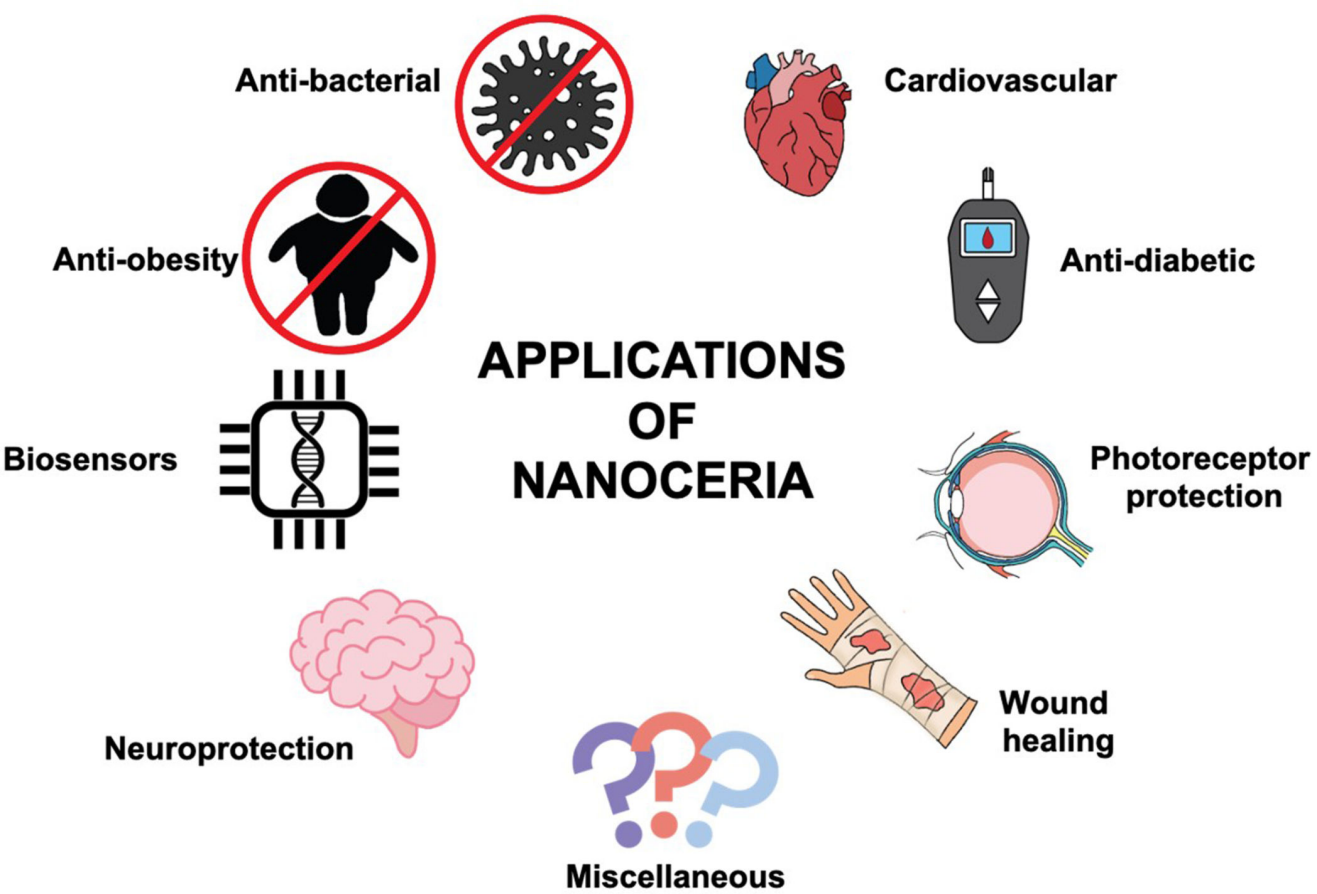
A schematic illustration of biomedical application for nanoceria.

The Ce^3+^ ions play a significant role in conferring reactive oxygen species (ROS) scavenging capabilities and other enzyme-like functions, making nanoceria effective antioxidants [15]. In addition to redox switching and oxygen vacancies in nanoceria, further scientific investigations are underway to elucidate their antioxidant activity. For instance, research by Celardo et al. demonstrated that modifying the redox state without affecting oxygen vacancies, such as doping with samarium, significantly reduced the antioxidant performance of nanoceria [20]. More recent studies by Wang et al. suggest that the antioxidant properties of nanoceria are linked to transient surface defect states (TSDSs) found in the electronic band structure of short-lived intermediate species [21]. Besides mimicking enzymes like superoxide dismutase (SOD) and catalase, nanoceria also utilizes non-catalytic chemical reduction pathways to neutralize superoxide ions and hydrogen peroxide, acting as reducing agents rather than catalysts [21]. This multifaceted mechanism underpins the extensive range of biomedical applications for nanoceria. The applications of nanoceria for biomedical applications are depicted in Figure 1.

### 2.1. Nanoceria Synthesis for Biomedical Use

The therapeutic performance of nanoceria is highly sensitive to its synthesis route, which determines key physicochemical properties such as size, shape, crystallinity, surface charge, and Ce^3+^/Ce^4+^ ratio. Several synthesis methods are commonly used, each producing nanoceria with distinct characteristics:

- Precipitation and Hydrothermal Methods: These traditional techniques are scalable and can yield crystalline nanoceria with controlled morphology but often result in particle size variation and agglomeration.
- Green/Bio-Directed Synthesis: Utilizing plant extracts, fungi, or polymers, this eco-friendly approach reduces toxicity concerns but offers limited control over particle uniformity.
- Oxidation and Sonochemical Methods: These methods offer rapid synthesis with smaller particle sizes, although reproducibility may be a challenge without tight control of reaction conditions.
- Microwave-Assisted and Combustion Methods: These allow rapid energy input and high yield, producing small, uniform nanoparticles, but may affect redox balance depending on precursors used.
- Microemulsion and Sol–Gel Methods: These techniques offer excellent control over size and shape but are more complex and sensitive to the reaction environment.

**Figure 2.**
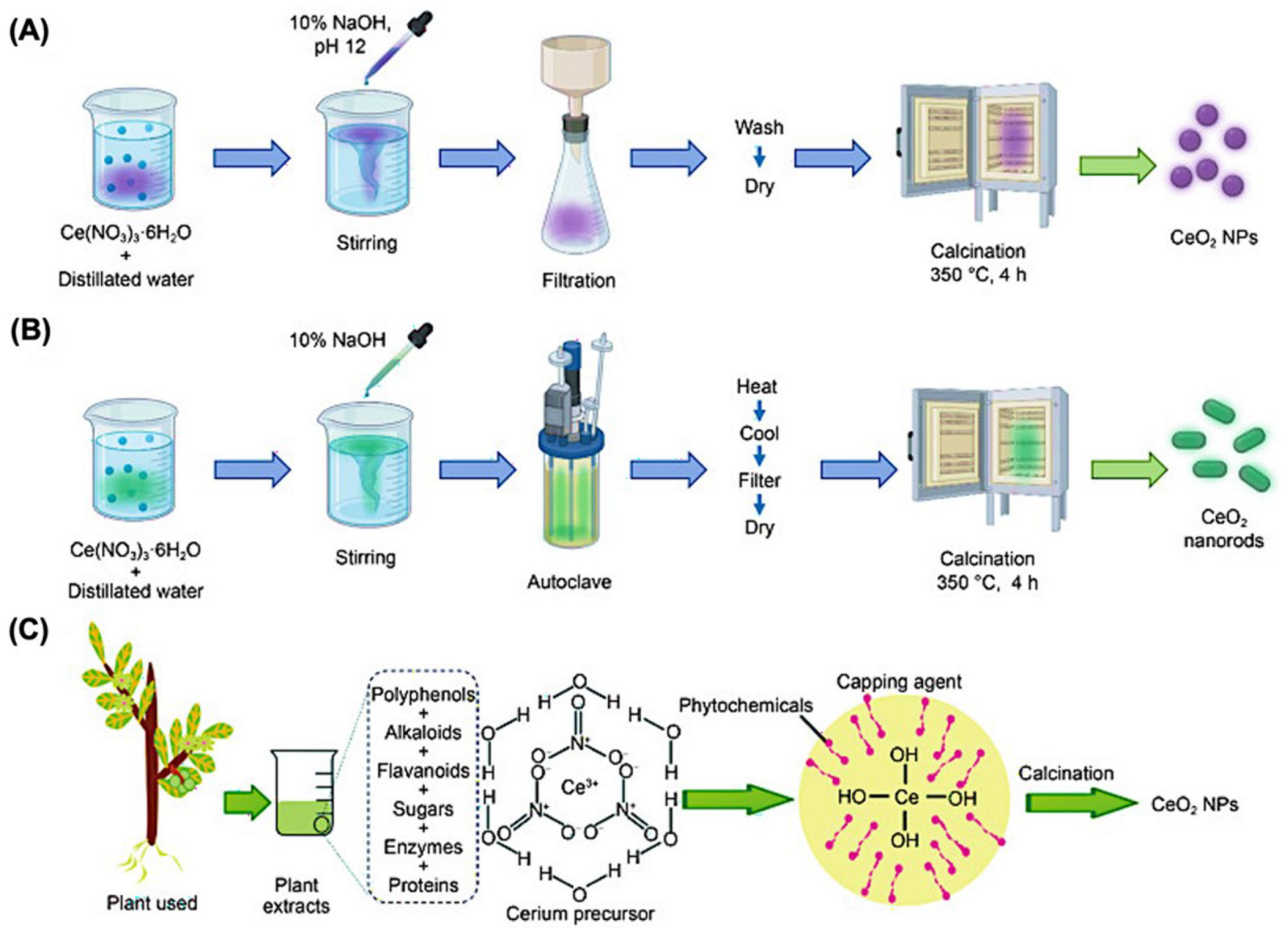
A schematic representation of the nanoceria synthesis through various methods. (**a**) The conventional precipitation method is commonly used for producing CeO_2_ NPs. (**b**) Nanorod-shaped CeO_2_ structures are typically obtained using a solution-phase hydrothermal technique. (**c**) The green synthesis approach can be used, where plant extracts serve as natural reducing and stabilizing agents in the formation of CeO_2_ NPs. Reproduced with permission from [22]. Copyright 2024, Springer publishers.

The lack of standardized protocols across these methods leads to discrepancies in biological behavior, especially in terms of redox activity, cellular uptake, toxicity, and therapeutic efficacy. For example, nanoceria with a higher Ce^3+^ content and minimal agglomeration demonstrates better antioxidant capacity and improved compatibility with immune cells and tissue scaffolds. To enable potential clinical translation, it is essential to develop reproducible and scalable synthesis approaches that produce nanoceria with well-defined, application-specific characteristics. Standardization will not only improve consistency across studies but also ensure safety, efficacy, and regulatory compliance for future biomedical applications. A pictorial representation of various synthetic routes for nanoceria preparation is given below (Figure 2).

### 2.2. Cellular Uptake and Localization of Nanoceria in Organs

Understanding how nanoceria is absorbed and distributed within biological systems is essential, as these particles play significant roles in applications like drug delivery, imaging, and cancer treatment. The physical characteristics of nanoparticles, including size, shape, and surface features, strongly influence their cellular uptake [23]. It has been found that nanoparticles smaller than 70 nm enter cells more swiftly, with those under 10 nm being particularly effective for targeted drug delivery [24]. Surface modification with PEG or targeting ligands can enhance uptake efficiency, prevent particle aggregation, and extend blood circulation [25,26]. Factors such as surface coating, size, electrolyte levels, and environmental pH greatly affect nanoparticle aggregation in biological fluids and overall systemic targeting efficiency.

Nanoparticles can cross cellular membranes through active or passive means. While lipid-based nanoparticles may enter cells via passive diffusion involving lipid interactions with the membrane [27], most nanoparticle uptake involves energy-dependent processes. This active uptake involves invaginations of the plasma membrane, forming vesicles through endocytosis or phagocytosis. The endocytic internalization pathways are diverse, including clathrin-mediated, caveolin-mediated, and other types of endocytosis, as well as micropinocytosis [28]. Tracking these uptake processes often relies on labeling nanoparticles with fluorescent dyes or radioactive tags, since nanoceria does not possess an intrinsic fluorescence [7,29].

**Figure 3.**
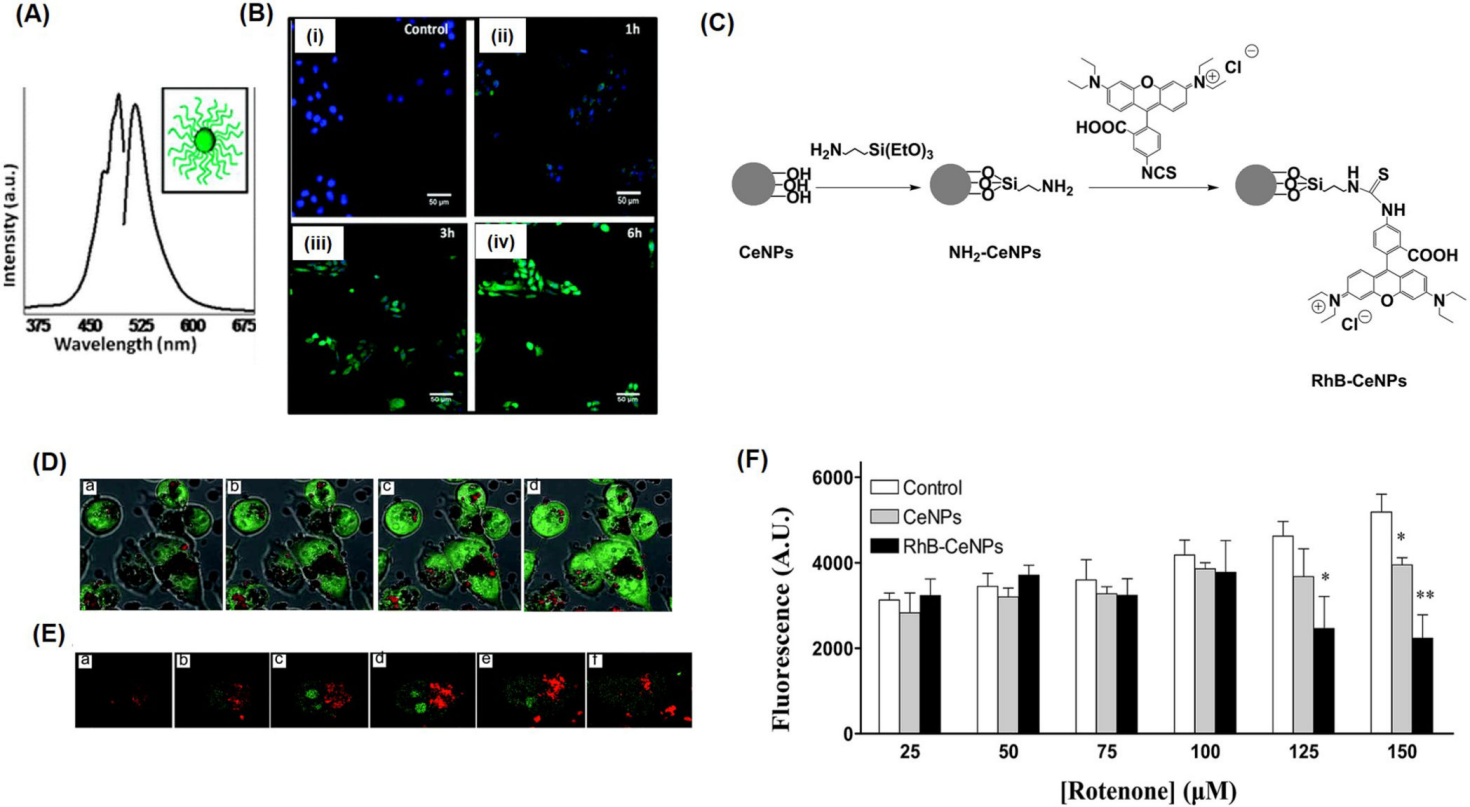
Fluorescently labeled nanoceria. (**A**) Emission spectra for CruzFluor fluorophore-labeled nanoceria. (**B**) Time-dependent cellular uptake of nanoceria observed within the first 1–3 h of incubation. Cells were treated with nanoceria in PBS for 1 h (**ii**), 3 h (**iii**), and 6 h (**iv**). Nuclei were counterstained with DAPI. Control cells not exposed to nanoceria (**i**) exhibited no significant fluorescence signals. Scale bar 50 μm. (**C**) Synthetic route for conjugation of nanoceria with Rhodamine B. (**D**) Confocal microscopy analysis of RhB-nanoceria uptake in HeLa cells following 3 h incubation. Live-cell z-stack images (**a**–**d**) illustrate nanoparticle distribution. (**E**) Merged brightfield and fluorescence images reveal cytoplasmic staining with N-acridine orange (NAO), emitting green fluorescence. (**E**) Single-cell views highlight green fluorescence from SYTO80-labeled nucleic acids, while the red signal corresponds to the intrinsic fluorescence of RhB-nanoceria. (**a**–**f**) represents live-cell z-stack images. (**F**) Evaluation of the impact of nanoceria and RhB-nanoceria (both at 20 μg/mL) on rotenone-induced reactive oxygen species (ROS) generation in HeLa cells. Bar graphs depict DCFH fluorescence intensity as a measure of ROS levels across varying concentrations of rotenone. Data are presented as mean ± SEM from three independent experiments (n = 3) and analyzed using one-way ANOVA. Statistical significance compared to untreated control cells is indicated as * *p* < 0.05 and ** *p* < 0.01. Reproduced with permission from [28,29]. Copyright 2010 and 2015, RSC publishers.

Self et al. demonstrated that fluorescently labeled nanoceria, with an average size of 3–5 nm, can localize in mitochondria, lysosomes, and the endoplasmic reticulum. The nanoceria were conjugated with CruzFluor fluorophores, and shortly after exposure to HaCat cells, fluorescence microscopy imaging revealed that the cells internalized the particles faster than adherent ones (Figure 3A,B). The uptake occurs mainly through clathrin-mediated endocytosis and involves localization within the cytoplasm, with no observed toxicity at imaging concentrations [28]. In another study, Nadezda et al. used nanoceria conjugated with rhodamine B (~10 nm) as a sensor for ROS and as an antioxidant in human cell lines (Figure 3C) [29]. The NPs showed enhanced fluorescence in the cytoplasm, indicating its ability to stain cytoplasm rather than mitochondria (Figure 3D,E). These dye-tagged particles demonstrated antioxidant activity and successfully detected oxidative stress via fluorescence, underscoring their potential as multifunctional probes combining therapy and diagnostics (Figure 3F).

Extensive research has explored how nanoceria distributes throughout the body, revealing that it tends to accumulate in organs associated with the mononuclear phagocyte system. Following intravenous injection, most of the nanoparticles (around 85–95%) are found in the liver and spleen, a trend consistent in both healthy and diseased animal models [30,31]. Minor accumulation is also noted in organs such as the kidneys, lungs, lymph nodes, ovaries, and bone marrow. Interestingly, the nanoceria dosage, particle shape, or dosing frequency appears to have little impact on their overall biodistribution [32]. The liver often serves as a long-term reservoir for nanoparticles, including nanoceria, and tissues like the kidneys, skeleton, and bone marrow gradually accumulate the particles over time. In a chronic inhalation study lasting 24 months, the lungs showed a maximum burden of 4.41 mg of CeO_2_ per lung, with minimal movement of particles less than 1% to other areas of the body [33]. Clearance from both the lungs and gastrointestinal tract is extremely limited. Furthermore, the nanoparticles can remain within cells and organs for extended periods, indicating slow elimination. Cellular uptake generally increases for up to 24 h after exposure, followed by a gradual decline [34].

### 2.3. Metabolism and Elimination of Nanoceria

The pharmacokinetic behavior of nanoparticles, including nanoceria, is markedly distinct from that of conventional small-molecule drugs. Unlike typical pharmaceuticals, nanoceria and other nanoparticles are rapidly cleared from circulation, exhibit minimal metabolic transformation, and are slowly eliminated from the body [35]. Clearance primarily occurs through two biological routes: hepatobiliary excretion, where nanoceria is processed by the liver and expelled via feces, and renal filtration, resulting in urinary elimination. Among these, liver-mediated clearance dominates, with studies reporting approximately a 60% reduction in hepatic content and a total body reduction of around 50% within 100 days following intravenous administration of nanoceria [31]. Notably, the highest excretion levels are observed within the first 24 h, as evidenced by elevated cerium levels in fecal matter.

Particle size plays a critical role in clearance dynamics [36]. Nanoceria with a hydrodynamic diameter under 6 nm is more readily removed through the kidneys. In contrast, larger particles, such as those around 30 nm, tend to persist in tissues for extended periods, showing negligible clearance over three months. This size-dependent degradation enhances the clearance for smaller particles. For example, a study involving 3 nm nanoceria demonstrated progressive cerium reduction across various organs, with the liver showing the most substantial decline. In the study, the ultra-small nanoceria (~3 nm) is more susceptible to dissolution, particularly at neutral or acidic pH, facilitating the formation of Ce^3+^ ions that can be excreted renally. However, following oral exposure, limited systemic absorption occurs, with most of the cerium detected in feces, though trace amounts may accumulate in internal organs. Within the liver, both hepatocytes and Kupffer cells are capable of uptaking and processing nanoceria, influencing its biodistribution and excretory fate [37,38].

### 2.4. Immunogenicity of Nanoceria

Understanding the immune interactions of nanoceria is essential for advancing their biomedical usages. These nanoparticles are generally recognized for their minimal toxicity and their ability to mitigate cellular damage, making them attractive candidates for therapeutic use. Particularly, nanoceria with dimensions smaller than 5 nm exhibit notable catalytic behavior, which is advantageous in both promoting and regulating inflammatory responses [39]. Research indicates that nanoceria can influence immune activity, often exhibiting protective, anti-inflammatory effects [40]. One of the key mechanisms involves the neutralization of reactive oxygen species (ROS), thereby functioning as an antioxidant within biological systems. In some experimental models, nanoceria has even been observed to enhance the immune response to vaccines, such as the influenza vaccine, especially when coated with specific stabilizers like citrate, which plays a role in improving immunogenicity. In general, nanoparticles that are poorly soluble, larger in size (>50 nm), or have positively charged or hydrophobic surfaces are more likely to trigger immune detection. In contrast, smaller, water-soluble particles with negative surface charges (<10 nm), like nanoceria, tend to evade immune recognition. Although initial studies support the potential of nanoceria in immunotherapy, more comprehensive research is required to clearly define its immunomodulatory properties [41].

### 2.5. Factors Affecting Different Activities of Nanoceria

The catalytic efficiency and functional roles of nanomaterials are strongly influenced by their physical and chemical properties, including dimensions, morphology, and surface composition. In a study conducted by Lord et al., nanoceria were synthesized in sizes ranging from 3 to 94 nanometers to assess their capability to neutralize ROS in human monocytes and macrophages [42]. The findings indicated that robust antioxidant activity appeared to be independent of both particle size and oxygen vacancy concentration. Complementing this, Vassie and coworkers explored how the particle size of nanoceria affected cellular uptake, internalization, and ROS scavenging in cancer cells, concluding that larger particles (~94 nm) had enhanced ROS-neutralizing performance [34]. They also discovered that nanoceria enter cells through energy-dependent mechanisms involving clathrin-mediated endocytosis, caveolae, and other non-specific routes.

Lee and colleagues examined ultrasmall nanoceria (~4 nm) and noted their potent antioxidant capabilities, which were further modulated by the thickness of polymer coatings [43]. Thinner coatings promoted more rapid interaction between Ce^3+^ ions and hydrogen peroxide. Meanwhile, Patil’s group analyzed the impact of surface charge on protein binding and cellular uptake, revealing that positively charged nanoparticles had a higher affinity for protein adsorption, whereas negatively charged ones demonstrated more efficient entry into A549 cells [44]. In related work, Celardo demonstrated that nanoceria can mitigate apoptosis triggered by various toxic agents in a dose-responsive manner, with protective effects closely tied to the presence of Ce^3+^ ions and their ROS-scavenging function [20]. Research by Asati et al. emphasized that negatively charged nanoceria were preferentially internalized by cancer cells and that their cytotoxic impact was enhanced when the particles accumulated in lysosomes instead of remaining in the cytosol [45].

Further insights into the activity of nanoceria revealed that enzyme-mimetic behavior depends on their surface crystal facets. Yang et al. synthesized nanocubes and nanorods with consistent Ce^3+^/Ce^4+^ ratios and oxygen vacancy levels but differing exposed facets; nanocubes with 100 surfaces exhibited greater peroxidase-mimicking activity, while nanorods with 110 facets showed stronger superoxide dismutase (SOD)-like activity [46]. Additionally, Gubernatorova’s team explored the potential of enhancing cerium oxide antioxidant properties through doping. Incorporating europium (Eu) into the cerium oxide structure increased oxygen vacancy levels, leading to improved ROS mitigation, particularly in a model of intestinal injury caused by ischemia–reperfusion [47].

## 3. Enzyme-Mimetic Properties of Nanoceria

X-ray photoelectron spectroscopy (XPS), Fourier-transform infrared spectroscopy (FTIR), and ultraviolet-visible (UV–Vis) analyses demonstrated that treating nanoceria with hydrogen peroxide (H_2_O_2_) reduces the proportion of Ce^3+^ relative to Ce^4+^ ions and indicates the formation of peroxyl groups on the nanoparticle surface [48–50]. The primary mechanism for neutralizing reactive oxygen species (ROS) by nanoceria relies on redox reactions and oxygen exchange occurring at their surface. The simultaneous presence of Ce^3+^ and Ce^4+^ allows these nanoparticles to catalytically interact with superoxide anions (O_2_^−^) and H_2_O_2_ by oxidizing Ce^3+^ and reducing Ce^4+^ [51,52], thereby scavenging a wide array of ROS types [53,54].

**Scheme 1.**
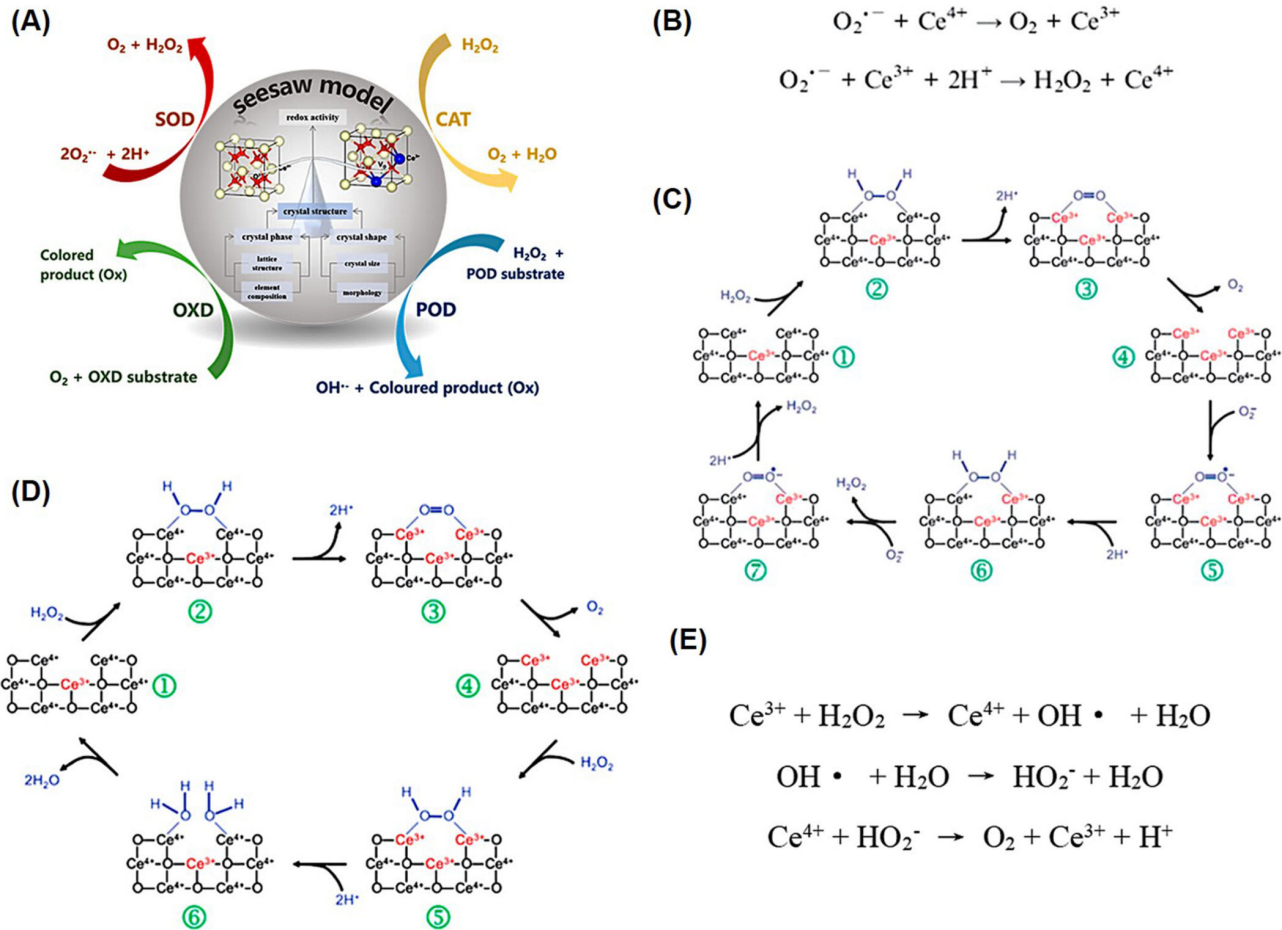
(**A**) A schematic representation of a dynamic seesaw-like model of nanoceria and different enzyme-mimicking activities by ceria-based nanozymes. (**B**) Schematic representation of the SOD-mimetic mechanism of nanoceria. (**C**) The mechanism involves several redox steps at the nanoparticle surface: ① Oxygen vacancies present on the nanoceria surface act as active sites; ② H_2_O_2_ binds to surface Ce^4+^ sites; ③ This interaction leads to proton release and electron transfer, reducing Ce^4+^ to Ce^3+^; ④ Molecular oxygen (O_2_) is subsequently released from the reduced vacancy site; ⑤ A superoxide (O_2_^−^) radical binds to the exposed oxygen vacancy; ⑥ The radical undergoes dismutation to form H_2_O_2_, which is released from the surface. ⑦ A second superoxide anion binds and completes the redox cycle, mimicking native superoxide dismutase (SOD) activity by converting reactive oxygen species (ROS) into less harmful products. (**D**) Illustrated mechanisms of catalase-like activity by nanoceria: The steps involved in the oxidation reactions follow the same order as shown in Scheme 1C (steps ① to ④). Step ⑤ represents the interaction of hydrogen peroxide (H_2_O_2_) with a site containing two Ce^3+^ ions, while step ⑥ denotes the uptake of two protons during the process. (**E**) Reaction pathway for peroxidase catalysis using nanoceria. Reproduced with permission from [48]. Copyright, 2023, American Chemical Society.

While much research has focused on the ROS-scavenging capabilities of nanoceria, particularly their reactivity toward hydrogen peroxide, studies quantifying oxygen transport and specific ROS in biological contexts remain limited. Evidence shows that exposure to H_2_O_2_ leads to the oxidation of surface Ce^3+^ ions into Ce^4+^ and the generation of superoxide (O_2_^−^) complexes on the nanoparticle surface, as verified by FTIR and XPS data [14]. Changes in the XPS spectra after H_2_O_2_ treatment confirm the dynamic redox behavior [49]. Furthermore, the detection of O_2_^2−^ stretching vibrations at 852 cm^−1^ in FTIR spectra after H2O2 exposure supports the hypothesis of superoxide adsorption onto the surface of nanoceria, highlighting a surface-bound mechanism of ROS interaction. The catalytic behavior of nanoceria is largely governed by the dynamic redox cycling between Ce^3+^ and Ce^4+^ ions. This reversible redox interplay acts like a seesaw, enabling fine-tuning of their enzyme-mimicking activities. Typically, nanoceria exhibits four major types of enzyme-like functions: superoxide dismutase (SOD)-like, catalase (CAT)-like, oxidase (OXD)-like, and peroxidase (POD)-like activities. A general schematic for the enzyme-mimetic properties of nanoceria is represented in Scheme 1A.

### 3.1. Superoxide Dismutase (SOD)-like Activity

Superoxide dismutase (SOD) is a key antioxidant enzyme that converts harmful superoxide radicals (O_2_•^−^) into oxygen and hydrogen peroxide (H_2_O_2_). Despite its biological importance, natural SOD has clinical limitations due to high production costs and a short half-life. Interestingly, nanoceria exhibit SOD-like activity, largely attributed to the redox cycling between Ce^3+^ and Ce^4+^ ions. Caputo et al. proposed that this redox switch enables nanoceria to mimic SOD by facilitating electron transfer. However, the slower transfer from Ce^3+^ to O_2_•^−^ appears to limit the reaction rate, meaning higher Ce^3+^ content improves efficiency [55] (Scheme 1B,C). Heckert et al. confirmed via EPR that SOD-like activity increases with the Ce^3+^/Ce^4+^ ratio, which can be tuned by reducing nanoparticle size [56]. Baldim’s research further demonstrated that smaller nanoceria (4.5–28 nm) with higher Ce^3+^ content showed enhanced catalytic behavior following a Langmuir isotherm [57].

Doping nanoceria with low-valence ions, such as europium, boosts oxygen vacancies and Ce^3+^ levels, further enhancing enzymatic activity and enabling fluorescence-based biosensing [58]. Mechanistically, nanoceria either follow a dismutation pathway or an oxygen vacancy-mediated process where superoxide is adsorbed, and Ce(III) transfers electrons, generating H_2_O_2_. In turn, H_2_O_2_ participates in the redox cycling of Ce ions, allowing continuous superoxide scavenging. Although current evidence supports the positive role of Ce^3+^ in SOD-like activity, the detailed redox interactions with H_2_O_2_ remain poorly understood and warrant further experimental and theoretical investigation.

### 3.2. Catalase (CAT)-like Activity

Nanoceria exhibits catalase-like activity by breaking down H_2_O_2_ into water and oxygen, helping to reduce oxidative stress. This activity is influenced by the Ce^4+^/Ce^3+^ ratio, with higher Ce^4+^ levels generally enhancing the catalytic function. Studies suggest that Ce^4+^ plays a crucial role in electron transfer during H_2_O_2_ decomposition, potentially making it the rate-limiting step in the reaction [52] (Scheme 1D). Although further studies are needed to clarify this mechanism, experimental data show that CeO_2_ with high Ce^4+^ content performs more effectively in biological environments than CeO_2_ rich in Ce^3+^ [59]. For instance, in liver cells where natural catalase is inhibited, nanoceria significantly lowered H_2_O_2_ levels, indicating their therapeutic promise [59,60]. The accepted model for catalysis involves a two-step redox process between Ce^4+^ and Ce^3+^ during H_2_O_2_ interaction [61]. However, some studies challenge this by suggesting the reaction may not rely on localized Ce^3+^ but instead involve broader electron delocalization [21]. The dynamic behavior of oxygen vacancies further complicates pinpointing the exact reaction sites. Given these complexities, more in-depth mechanistic studies are essential to optimize enzymatic nanoceria for biomedical applications.

### 3.3. Oxidase (OXD)-like Activity

Oxidase (OXD) enzymes play a vital role in human physiology by catalyzing the oxidation of substrates, including amino acids, amines, and alcohols, using molecular oxygen. This process produces hydrogen peroxide or water, and sometimes superoxide radicals, often accompanied by a visible color change, making OXD enzymes useful for biosensing applications. Despite their importance, reports of nanomaterials with strong oxidase-like activity remain limited. Nanoceria exhibit enzyme-like behaviors due to their unique redox “seesaw” mechanism, which is influenced by their size, shape, and surface chemistry (Scheme 1A). While nanoceria typically shows SOD and CAT activities depending on its Ce^3+^/Ce^4+^ ratio, studies have demonstrated that modifications like dextran or poly(acrylic acid) coatings enhance its OXD-like activity, especially under acidic conditions [62,63]. For example, acidic pH improves the ability of nanoceria to fully oxidize ampliflu, unlike the partial oxidation observed at neutral pH. Fluoride capping further boosts this activity by enhancing substrate binding, preventing product inhibition, and promoting electron transfer [64]. Mechanistic studies show that under acidic conditions, nanoceria adsorbs oxygen, facilitating its reduction to superoxide (O_2_•–), which is subsequently converted to H_2_O_2_ and ultimately to hydroxyl radicals via a Fenton-like reaction. This explains the strong oxidase activity of nanoceria in acidic environments. Leveraging this, researchers have developed sensitive cancer biomarker detection methods by nanoceria, demonstrating their promise for diagnostics in clinical and point-of-care settings [62,65].

### 3.4. Peroxidase (POD)-like Activity

Peroxidases (PODs) are crucial enzymes in the antioxidant defense system, catalyzing the oxidation of various substrates using hydrogen peroxide as an oxidizing agent. This diverse group includes enzymes like glutathione peroxidase, which mitigates oxidative stress by neutralizing intracellular ROS, and myeloperoxidase, known for its role in immune defense against pathogens. Horseradish peroxidase (HRP) is widely employed in clinical diagnostics due to its ability to catalyze chromogenic reactions with specific substrates. Recently, increasing attention has been directed toward inorganic nanomaterials that emulate peroxidase activity. Nanoceria and their composites have shown significant promise [48,66–68]. For instance, Mn(II)/Ce composites have enabled the sensitive detection of hydrogen peroxide and glucose [69], while Ce-SrMOF-based sensors have been developed for assessing antioxidant levels in the saliva of lung cancer patients [70]. These advances highlight the need to better understand the mechanisms underlying their catalytic function.

Despite both utilizing H_2_O_2_, catalase and peroxidase differ mechanistically: catalase promotes H_2_O_2_ disproportionation into water and oxygen, whereas ceria-based peroxidase mimics decompose H_2_O_2_ into reactive radicals like hydroxyl and superoxide species, which then oxidize target substrates (Scheme 1E). Research by Heckert et al. revealed that Ce^3+^ surface sites play a vital role in H_2_O_2_ adsorption and radical generation during these reactions [71]. Furthermore, the enzyme-like activity of nanoceria correlates with its Ce^3+^ content and acidic conditions, enhancing hydroxyl radical production as evidenced by DNA relaxation assays. Building on this, Liu and colleagues developed porphyrin-modified ceria nanorods with superior POD-mimetic properties [8]. Their study demonstrated that porphyrin facilitated electron transfer to nanoceria under light activation, reducing recombination losses and enhancing the formation of reactive oxygen species. This synergistic approach opens new pathways for developing highly efficient nanozymes.

## 4. Biomedical Applications for Nanoceria

Nanoceria exhibits a wide range of biomedical uses, including roles in antioxidant therapy [72,73], cancer treatment [74], and antimicrobial action [75,76], as well as in drug and gene delivery [77], biosensing [78,79], medical imaging [80], and controlling inflammation [8,81]. Their favorable biocompatibility and selective cytotoxicity in diseased cells make them highly attractive for clinical use. Moreover, these nanoparticles have shown considerable potential in promoting wound repair [82,83] and tissue regeneration [84], primarily through their regulation of oxidative stress and inflammatory responses. In this review, we highlight the state of the art in using nanoceria for various biomedical applications related to their antioxidant and anti-inflammatory properties.

### 4.1. Nanoceria for Inflammation Studies

#### 4.1.1. Rheumatoid Arthritis

Nanoceria has emerged as a promising alternative to traditional non-steroidal anti-inflammatory drugs (NSAIDs). While conventional agents such as ibuprofen, naproxen, and corticosteroids are widely used to manage both acute and chronic inflammatory conditions, including minor injuries and severe diseases like asthma, they are often limited by significant side effects [85]. In contrast, nanoceria offers several therapeutic benefits, including selective localization at inflammation sites, redox-based catalytic activity, and prolonged retention in affected tissues [49,82]. Importantly, research suggests that nanoceria induces minimal toxicity, enhancing its appeal as a safer option. Its efficacy is particularly notable in addressing chronic inflammation, which can persist for extended periods and lead to tissue damage due to unresolved immune signaling. Unlike acute inflammation, chronic conditions often persist without resolution, causing a range of diverse symptoms that depend on the affected tissue. As existing treatments struggle to address long-term inflammatory diseases, nanoceria represents a compelling avenue for future therapeutic development. Nanoceria has shown significant potential in controlling inflammation across various disease conditions by lowering the levels of ROS. Its strong anti-inflammatory action stems from its ability to neutralize free radicals effectively. Additionally, nanoceria exerts its therapeutic effects by influencing key inflammatory pathways, including the suppression of NF-κB, IL-6, and IL-8, while also enhancing the expression of antioxidant enzymes like superoxide dismutase and catalase [86,87].

Rheumatoid Arthritis (RA) is a chronic autoimmune disease marked by persistent inflammation and ROS production. Nanoceria shows promise in alleviating pain and reducing ROS in RA. Zhang et al. developed silver-modified ceria nanoparticles loaded with celastrol (Ag-CeNP@Cel), which enhanced celastrol’s solubility and scavenged ROS, promoting M1-to-M2 macrophage transition [88]. This reduced inflammation and improved the RA microenvironment. The nanoparticles, tagged with cyanine dye for imaging, showed 4 times higher fluorescence in inflamed versus non-inflamed legs in arthritic mouse models.

**Figure 4.**
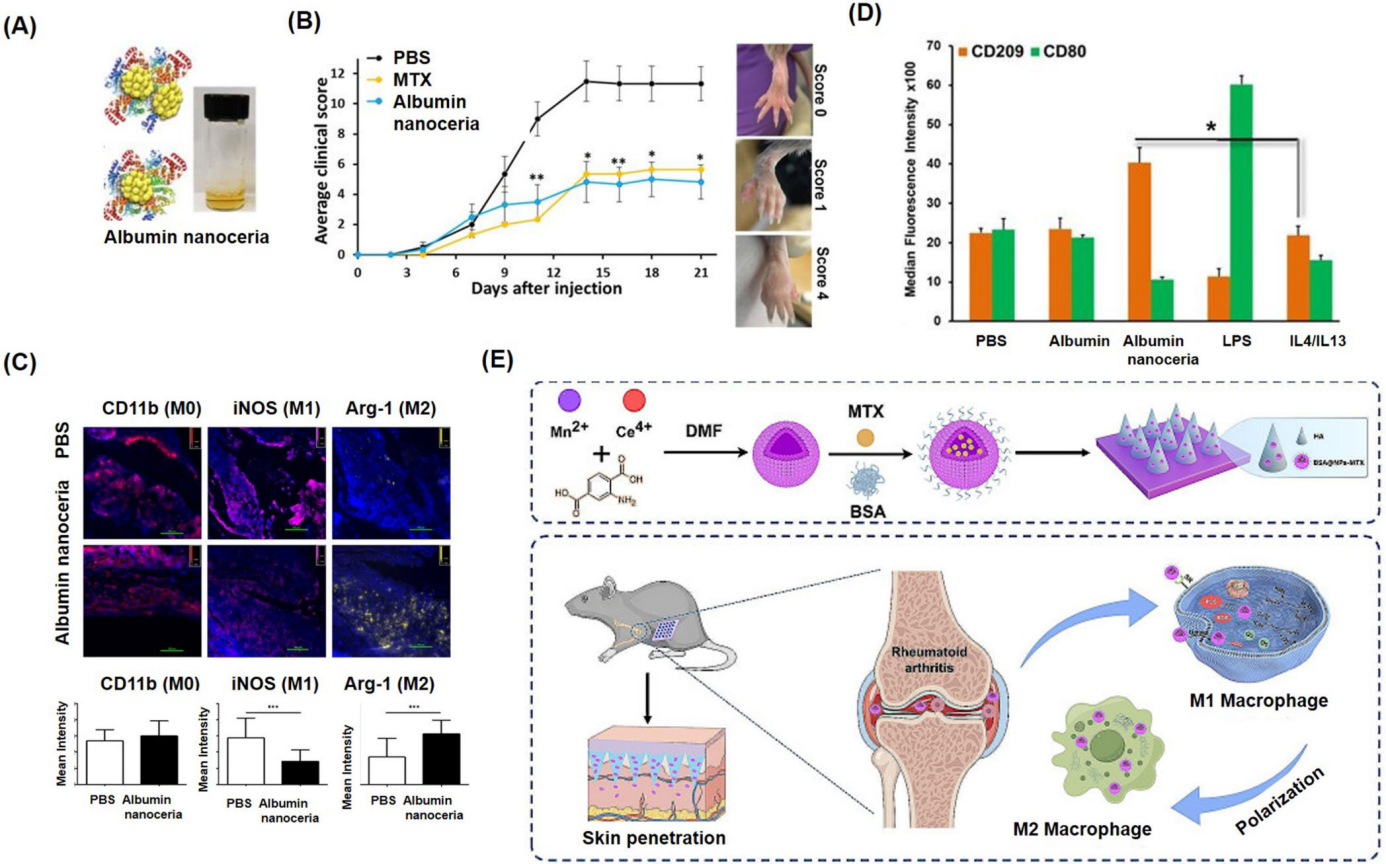
Application of nanoceria for Rheumatoid Arthritis. (**A**) A pictorial representation for the albumin-nanoceria and its visual appearance. (**B**) Assessment of the therapeutic efficacy of albumin-nanoceria in CIA mice through clinical scoring over a 3-week period (N = 6). Mice treated with albumin-nanoceria (50 μL, blue line) exhibited an average clinical score of 4.8 ± 1.1, comparable to the methotrexate (MTX) group (50 μL, yellow line), and significantly lower by approximately 2.4-fold than the PBS control group (black line). The inset displays representative images of paw inflammation used for scoring. Error bars represent the standard error of the mean (SEM); statistical significance indicated as * *p* < 0.05, ** *p* < 0.005 versus PBS control. (**C**) Fluorescence images of tissue sections from PBS and albumin-nanoceria-treated mice show high macrophage infiltration (CD11b, red) in inflamed paws (DAPI, blue). Albumin-nanoceria treatment reduced pro-inflammatory M1 macrophages (iNOS, pink) and increased anti-inflammatory M2 macrophages (Arg-1, yellow). The lower panel represents quantified fluorescence signals of CD11b, iNOS, and Arg-1 across 25 ROIs (five images from two mice per group; error bars = SD; *** *p* < 0.0005). (**D**) Mean fluorescence intensity data for THP-1 cells treated with albumin-nanoceria. The albumin-nanoceria treated cells showed lower CD80 than samples treated with IL4/IL13 (N = 3, error bars = SD, *p*-value: * *p* < 0.05). (**E**) A schematic representation and therapeutic application of manganese dopped nanoceria for RA. Reproduced with permission from [49,89]. Copy right 2020, Ivy Spring Publishers. 2024, Springer publishers.

Kalashnikova et al. from our group demonstrated that RA can be effectively treated using inflammation-targeting albumin-nanoceria [49]. Small, Ce^3+^-rich nanoceria were synthesized on an albumin substrate via biomineralization (Figure 4A). Albumin, a natural blood protein that accumulates at inflammation sites, provided stability, biocompatibility, and safety to the nanoceria. In a collagen-induced arthritis mouse model, nanoceria treatment significantly reduced paw inflammation, showing efficacy comparable to methotrexate (MTX), a conventional antirheumatic drug (Figure 4B). Immunofluorescence revealed elevated Arg-1 (anti-inflammatory M2 marker) and reduced iNOS (pro-inflammatory M1 marker) in nanoceria-treated tissues, though macrophage presence (CD11b) remained high in both PBS- and nanoceria-treated mice (Figure 4C). In vitro, flow cytometry of Raw264.7 and THP-1 cells confirmed nanoceria’s immunomodulatory role, promoting a shift from pro- to anti-inflammatory phenotypes of macrophages (Figure 4D). In another study, Xia et al. used manganese-doped nanoceria to treat RA [89]. Manganese enhanced ROS scavenging and served as an MTX nanocarrier. Nanoparticles, coated with BSA and incorporated into hyaluronic acid microneedles (Figure 4E), showed strong ROS reduction in macrophages at 25 μg/mL, confirmed by flow cytometry and immunofluorescence analysis.

RA is characterized by synovial inflammation driven by pro-inflammatory M1 macrophages. Targeting the M1/M2 macrophage imbalance exacerbated by hypoxia and elevated ROS in the RA synovium is a promising therapeutic approach. Kim et al. developed manganese ferrite–ceria nanoparticle-anchored mesoporous silica nanoparticles (MFC-MSNs) to simultaneously scavenge ROS and generate oxygen, promoting M1-to-M2 macrophage polarization [90]. In the work, MFC-MSNs neutralize hydroxyl radicals produced during the manganese ferrite-mediated Fenton-like reaction. Intra-articular injection of MFC-MSNs in RA rat models alleviated hypoxia, inflammation, and joint damage. MTX-loaded MSNs further improved therapeutic outcomes through sustained drug release. Separately, Lin et al. reported R-dihydrolipoic-acid-stabilized cerium-modified gold nanoclusters (~3.4 nm) that rapidly normalized cytokine levels and suppressed B cell memory responses in collagen-induced arthritis models [91]. These nanoclusters outperformed standard RA drugs, highlighting their potential for treating advanced RA by targeting both oxidative stress and immune dysregulation.

#### 4.1.2. Nanoceria for Pancreatitis

Chronic pancreatitis, though less frequently discussed, is a devastating and life-threatening condition characterized by irreversible pancreatic damage, excessive extracellular matrix (ECM) deposition, and impaired pancreatic function. A major complication of this chronic disease is pancreatic cancer, and individuals affected by chronic pancreatitis typically experience a significantly reduced lifespan. Currently, there are no curative treatments available; therapies merely aim to slow disease progression without halting or reversing it. However, promising research by Godgu and colleagues demonstrates that nanoceria may offer a novel therapeutic approach [92]. In their study, ~100 nm nanoceria particles were synthesized and administered to animal models of chronic pancreatitis. The treatment significantly reduced inflammation by lowering levels of key inflammatory markers, such as NF-κB, and inhibited pro-fibrotic signaling pathways. Notably, nanoceria treatment led to reduced ECM accumulation and endoplasmic reticulum (ER) stress. These results indicate that nanoceria not only alleviates inflammation but may also substantially slow or even halt disease progression. This groundbreaking approach holds great potential as a transformative therapy for chronic, irreversible pancreatic disorders, offering patients improved outcomes and enhanced quality of life. In a separate study, Luo and colleagues demonstrated that utilizing a combination of calcium-binding agents and cerium-based nanozymes carrying catalase effectively reduced excessive ROS and alleviated mitochondrial damage, resulting in comprehensive anti-inflammatory benefits [93]. This nanotherapeutic system also helped restore disrupted autophagic processes and reduced endoplasmic reticulum stress in the pancreas, aiding the recovery of damaged acinar cells. On a mechanistic level, treatment with the nanoplatform corrected metabolic disruptions within pancreatic tissue and suppressed key inflammatory signaling pathways involved in the progression of pancreatic inflammation.

### 4.2. Nanoceria for Immunotherapy Applications

Nanoceria has been explored in specific immunotherapy applications, primarily as an immunomodulatory nanodrug [49]. One could argue that the treatment of rheumatoid arthritis with nanoceria and its associated modulation of macrophages from an M1 pro-inflammatory to an M2 anti-inflammatory phenotype constitutes a form of immunotherapy; however, in these applications, it is not being utilized to its full potential to affect the entire immune environment. Other populations of cells can be activated or deactivated, including T cells, B cells, and NK cells. Early evidence supports the claim that nanoceria can be used to modulate these cell populations, in addition to macrophages and monocytes. For diseases with complex immune phenotypes, such as endometriosis and cancer, this makes nanoceria an attractive option for treatment [7,94]. To thoroughly examine the use of nanoceria as an immunotherapy, applications of nanoceria beyond the simple tuning of macrophages must be explored and characterized.

#### 4.2.1. Role of Nanoceria in Tumor Microenvironment

Generally, tumors are remarkably difficult to treat due to the complex nature of the tumor microenvironment (TME). Tumors often have hypoxic cores with a lack of useable oxygen, which inhibits immune activity and prevents effective treatment [95]. Unfortunately, tumors are also known to have highly metabolically active exteriors with anti-inflammatory tumor-associated macrophages preventing an appropriate pro-inflammatory response to abnormal tumor cells [96,97]. Paradoxically, it has been shown that nanoceria can both be used to relieve tumor hypoxia by scavenging ROS and be used to generate ROS in tumor environments. This is due to the pH-responsive behavior of nanoceria, which allows for the scavenging of ROS in neutral or basic environments as commonly reported and the generation of ROS in acidic environments like those found in tumors as demonstrated by Wang et al. [98]. These smart NPs show a 3-in-1 action: pH responsiveness, controlled release of doxorubicin, and TME-responsive combination therapy. Glycol chitosan-coated nanoceria were loaded with doxorubicin, and for target specificity, the CXCR4 antagonist (AMD11070). Blocking CXCR4 allows for reducing the crosstalk between the TME and tumor cells, which leads to the inhibition of tumor metastasis. The study showed, under acidic pH conditions, tumor cell apoptosis and reduced tumor growth in vitro in human retinoblastoma (Rb) cells and in vivo in mouse genetic RbLox/lox p107+/− p130−/− (p107s) and human xenograft Rb models. Nanoceria has a dual effect, both protecting normal tissues from oxidative stress and unnecessary inflammation while triggering a much-needed inflammatory cascade in immunosuppressed cancers. As the hypoxic core of tumors does not have the same molecular environment as the acidic exterior, nanoceria instead restores oxygen to the area, allowing for greater efficacy of cancer therapies. Though research on nanoceria as an immunotherapy is still nascent, the particles hold enormous potential in a variety of immune modulated disease states beyond typical inflammatory conditions.

#### 4.2.2. Role of Nanoceria as an Anticancer Drug

Fernández-Varo et al. demonstrated that 4–5 nm nanoceria, synthesized via co-precipitation, exert strong antioxidant and anti-inflammatory effects in Wistar rats with hepatocellular carcinoma [99]. Treatment improved survival by reducing macrophage infiltration and phosphorylated ERK1/2 levels, key components of the Ras/MAPK pathway. Nanoceria also helped restore disrupted fatty acid metabolism, which is crucial for cancer cell growth. In a breast tumor model, nanoceria reduced oxidative and inflammatory markers like MDA, MPO, and nitric oxide. While promising, further studies are needed to clarify how nanoceria regulates lipid metabolism in disease.

**Figure 5.**
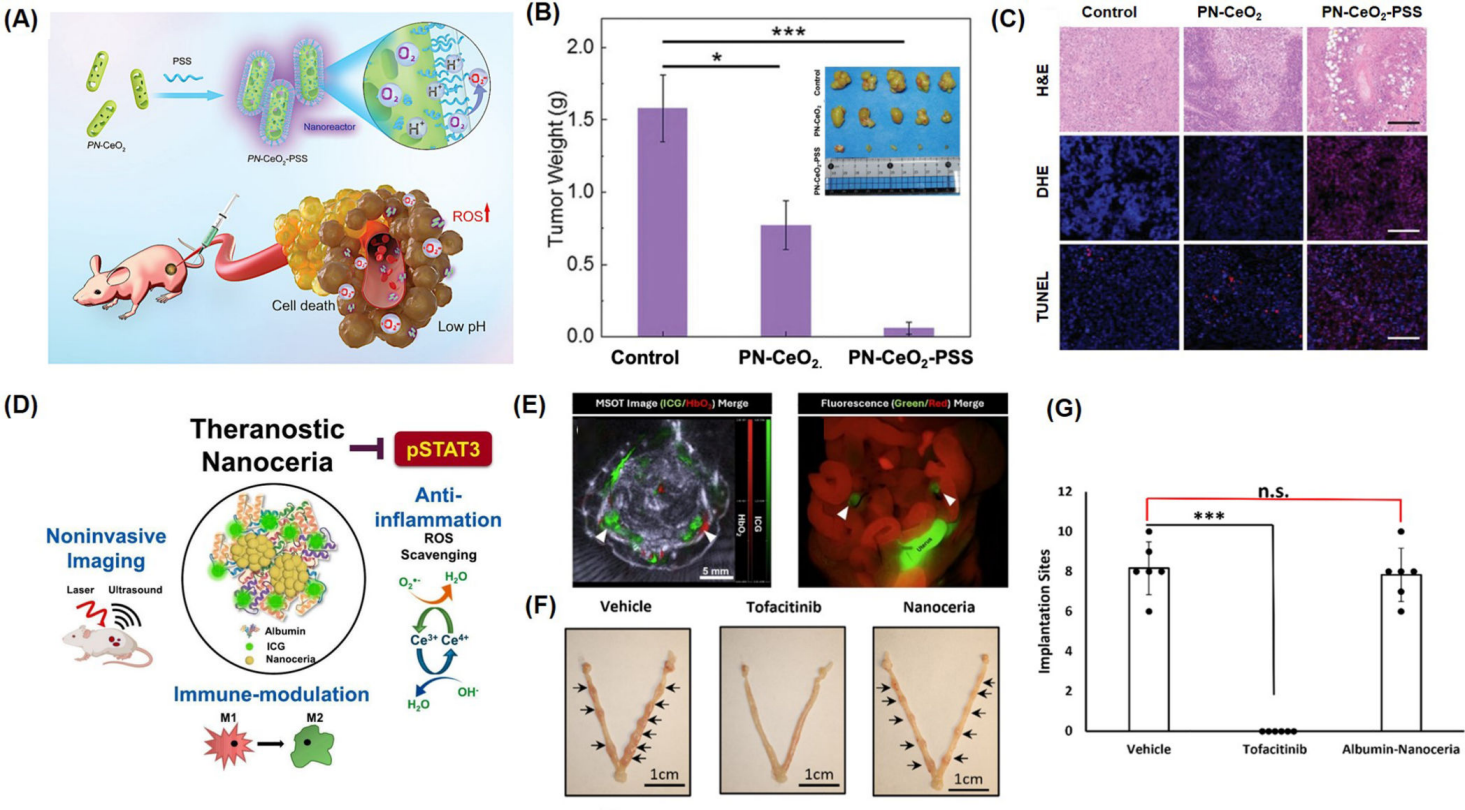
Immunotherapy applications of nanoceria. (**A**) A schematic representation and catalytic activity of porous nanoceria (PN-CeO_2_). (**B**) tumor volume from the non-treated and treated mice (PN-CeO_2_ and sodium polystyrene sulfonate (PSS) coated PN-CeO_2_) after 20 days of treatment. * *p* < 0.05 and *** *p* < 0.001. The inset represents the Ex vivo images for the tumor for the non-treated and treated mice. (**C**) H&E staining, ROS staining and TUNEL staining for pathological changes in tumor tissues of each group after the 20-day treatments. All scale bars are 100 μm. (**D**) A pictorial representation for the theranostic application of albumin-nanoceria with indocyanine green (ICG) for non-invasive detection of endometriosis. (**E**) represents the photoacoustic and fluorescence microscopic images for mice injected with albumin-Ce-ICG nanoparticles. The arrows indicate the endometrial lesions in the mice. (**F**) Representative ex vivo images of uteri with implantation sites (black arrows) of treatment groups at gestation day 5.5. (Scale bar = 1 cm). (**G**) A graphical representation for the implantation sites for non-treated, albumin-nanoceria-treated and Tofactinib-treated mice uterine horns. A statistically significant reduction in implantation sites between vehicle-treated and tofacitinib-treated mice was observed but not between vehicle- and albumin-nanoceria-treated cells, showing that albumin-nanoceria does not influence fertility (n = 6) (*** *p* <0.001). Reproduced with permission from [94,100]. Copyright 2020, Wiley publishers; 2025, Elsevier, respectively.

The therapeutic efficacy of nanoceria in cancer treatment has been explored across various preclinical models. In one study, Tian and colleagues demonstrated that intravenously delivered porous cerium oxide nanorods, synthesized using a hydrothermal technique, reduced tumor mass by 51.1% [100] (Figure 5A). This anti-tumor effect was further enhanced to 96.1% when the nanorods were coated with sodium polystyrene sulfonate (Figure 5B). Administration of nanoceria (~32 nm in size, carrying a surface charge of −26.3 mV intraperitoneally at a dose of 0.5 mg/kg to mice significantly inhibited the progression of WEHI164 tumors [101]. Additionally, a copper-doped version of nanoceria almost completely halted tumor growth, achieving 98.5% inhibition [102]. Reduced tumor cell proliferation was supported by the marked suppression of Ki67 expression in MDA-MB-231 tumors in mice [102] and hepatocellular carcinoma models in rats [99] after nanoceria treatment. Histological assessments using hematoxylin and eosin (H&E) staining indicated a greater presence of necrotic and apoptotic cells in treated tumor samples compared to controls (Figure 5C) [100]. Furthermore, TUNEL assays revealed extensive DNA fragmentation in nanoceria-treated tissues, highlighting increased levels of apoptosis (Figure 5C). Molecular analyses showed upregulation of pro-apoptotic genes such as Bax and caspase-3, alongside reduced expression of the anti-apoptotic gene Bcl-2. Elevated levels of reactive oxygen species (ROS), as indicated by dihydroethidium (DHE) staining, suggested that oxidative stress-induced apoptosis was a key mechanism of action (Figure 5C).

#### 4.2.3. Nanoceria for Endometriosis Treatment

Nanoceria is also being evaluated as a non-steroidal anti-inflammatory drug for endometriosis theranostics. Endometriosis is a chronic inflammatory disease affecting approximately 10% of reproductive-aged women globally, characterized by the growth of endometrial-like tissue outside the uterus [103,104]. Studies have identified a strong link between oxidative stress (OS) and the development of endometriosis, with abnormal endometrial angiogenesis also playing a central role in the disease’s progression. In a notable study by Chaudhury et al., nanoceria were successfully employed to alleviate symptoms of endometriosis [105]. These nanoparticles, characterized by their dual oxidation states (Ce^3+^ and Ce^4+^), function as efficient scavengers of free radicals such as superoxide and hydrogen peroxide. When administered intraperitoneally at a single dose of 0.5 mg/kg in a murine model, nanoceria significantly reduced the formation of endometrial lesions. This therapeutic effect was attributed to a decrease in oxidative stress markers, including ROS and lipid peroxidation, and an improvement in total antioxidant capacity, as well as suppressed angiogenesis, evidenced by lower levels of vascular endothelial growth factor and adrenomedullin. Compared to the commonly used antioxidant N-acetyl cysteine, administered at 250 mg/kg three times weekly for 15 days, nanoceria demonstrated superior outcomes. Additionally, the treatment helped improve oocyte quality, a key determinant of reproductive success in individuals with endometriosis.

Rahman and colleagues demonstrated the efficacy of nanoceria in the treatment of endometriosis, a disease characterized by complex immune interactions [94]. There are several differences between the immune profiles of healthy and diseased animals, including an increased prevalence of pro-inflammatory macrophages and T-cells as well as reduced NK cells and anti-inflammatory macrophages, which are present in endometrial lesions. Plausible treatments for endometriosis include common STAT and JAK inhibitors, which have been shown to reduce disease burden through limiting pro-inflammatory signaling. However, a major challenge present in the treatment of endometriosis is that lesions are phenotypically similar to normal uterine tissue. Unfortunately, the same inhibitors that are useful in reducing disease burden act on appropriate activation of STAT3 in the uterus, leading to many undesirable symptoms, including infertility [106]. For this reason, albumin-nanoceria was explored as a targeted STAT/JAK inhibitor and immunotherapy in endometriosis (Figure 5D). Nanoceria was able to have a distinct, targeted effect on endometrial lesions without affecting the uterus, as confirmed by fluorescence and photoacoustic signals from albumin-nanoceria conjugated with indocyanine green (ICG). These NPs enabled non-invasive detection of endometriosis lesions (Figure 5E). In model mice treated with albumin-nanoceria, there was a reduction in lesions comparable to the effect of the JAK inhibitor or Tofacitinib. The pSTAT3 (the activated form of STAT3) was reduced in the lesions of nanoceria-treated mice, and not only were M1 macrophages reduced and M2 macrophages increased, but T cells were reduced without an impact on NK cells. Fascinatingly, owing to its efficient targeting, nanoceria did not inhibit pregnancy in treated mice, whereas traditional therapeutics caused implantation failure (Figure 5F,G). These findings suggest that nanoceria can function as a STAT/JAK inhibitor, influencing the overall immune environment of lesions without compromising fertility, thereby positioning nanoceria as an excellent choice for treating endometriosis.

### 4.3. Theranostic Application of Nanoceria

**Figure 6.**
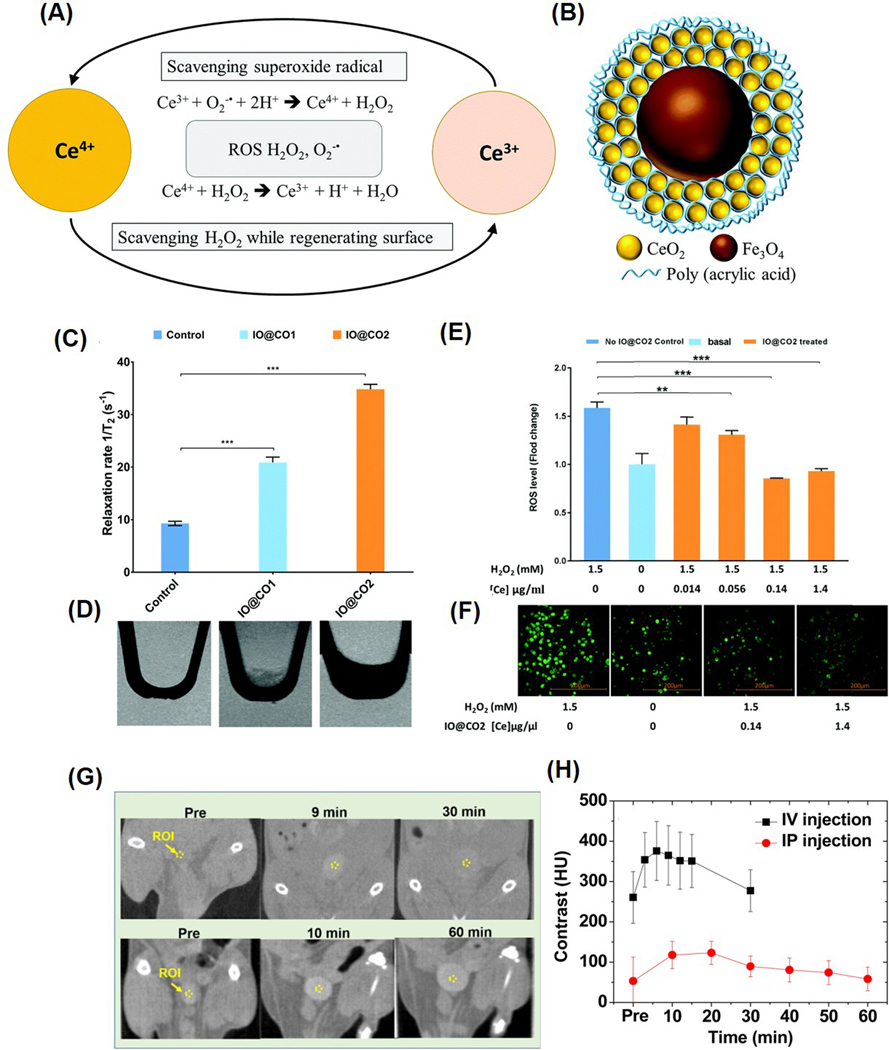
(**A**) A schematic showing the SOD-type activity for nanoceria. Cerium oxide exhibits regenerative antioxidant behavior by alternating between Ce^3+^ and Ce^4+^ states, scavenging superoxide radicals as Ce^3+^ and neutralizing H_2_O_2_ as Ce^4+^. (**B**) A structural model illustrates iron oxide–cerium oxide core–shell nanoparticles (IO@CO). (**C**) MRI relaxation profile for non-treated and IO@CO-treated macrophage cells. Treated cells with IO@CO1 and IO@CO2 showed a higher relaxation rate than untreated controls. *** *p* < 0.001. (**D**) MRI images revealed stronger dark signals in nanoparticle-treated cells, especially with IO@CO_2_, than untreated cells. (**E**) ROS scavenging ability of IO@CO NPs. ROS levels decreased significantly when cerium concentration reached 14 ng/100 μL or more than the control. ** *p* < 0.01, *** *p* < 0.001 (**F**) Fluorescence imaging confirmed reduced oxidative stress, as nanoparticle-treated cells emitted lower green fluorescence compared to H_2_O_2_-treated cells. Scale bar 200 μm. (**G**) In vivo CT images of the mice bladder before and after intravenous (IV) and intraperitoneal (IP) injections of an aqueous suspension sample of PAA-coated ultrasmall CeO2 nanoparticles at 70 kVp. The dotted circles at the bladder indicate the region of interest (ROI). (**H**) Contrast plots of the SNR-ROI of the bladder as a function of time. Reproduced with permission from [112,114]. Copyright, 2018, 2023, RSC publishing group.

Theranostic approaches in modern medicine offer a dual advantage by enabling simultaneous disease diagnosis and treatment through non-invasive imaging [107,108]. Nanoceria, especially when doped with gadolinium or surface-modified with targeting ligands, combine potent antioxidative therapeutic properties with enhanced diagnostic imaging capabilities, primarily magnetic resonance imaging (MRI) and computed tomography (CT) contrast enhancement [109–111]. Their size tunability, biocompatibility, ability to scavenge ROS, and targeting capability make them highly promising theranostic agents for personalized diagnosis, image-guided therapy, and monitoring of inflammatory and cancerous diseases. For instance, Wu et al. engineered Fe_3_O_4_/CeO_2_ core–shell nanoparticles that serve both diagnostic and therapeutic functions [112] (Figure 6A,B). In this system, iron oxide provided MRI contrast, while the cerium oxide shell delivered ROS-scavenging therapeutic benefits (Figure 6C,D). This multifunctional design not only facilitated MRI tracking and biodistribution analysis of the nanoparticles but also demonstrated high cellular uptake, a favorable Ce^3+^/Ce^4+^ ratio, and minimal cytotoxicity. These features make them promising tools for managing ROS-associated inflammatory disorders, including cardiovascular diseases, atherosclerosis, rheumatoid arthritis, and allergies (Figure 6E,F). The potential for targeted therapy was also discussed, highlighting the possibility of functionalizing these nanoparticles with antibodies or peptides targeting specific inflammatory markers, such as VCAM-1 and neutrophil cytosolic factor 1. Additionally, another study described magnetite-CeO_2_ nanoconjugates formed by interlinking iron oxide and nanoceria, coated with polyethyleneimine (PEI) and crosslinked using glutaraldehyde [113]. These 8 nm-sized particles exhibited enhanced antioxidant activity both in vitro and in vivo, possessing superparamagnetic properties and effectively neutralizing ROS. The combination of magnetic responsiveness and potent antioxidant activity positions these nanostructures as valuable candidates for advanced theranostic applications. In a similar study, Eriksson et al. showed that a 5 nm-sized nanoceria doped with gadolinium showed excellent T1 relaxivity with ROS scavenging property. The bioluminescence study showed an inhibitory effect of ROS in vivo with a higher concentration of Ce3+ at the nanoparticle surfaces [110]. At the same time, Saidi et al. showed the use of nanoceria for CT application for in vivo tumor tracking and treatment [114]. To improve stability and contrasting properties, the nanoparticles (1–3 nm in size) were coated with hydrophilic and biocompatible poly(acrylic acid) (PAA) and poly(acrylic acid-co-maleic acid). The nanoparticles showed distinctive CT contrast signals in the bladder, and interestingly, injection dose is ∼10 times less than those of standard iodine contrast agents (Figure 6G,H). A redox-active gadolinium-doped nanoceria was synthesized by Kolmanovich et al. [115]. Using polyelectrolyte, a layer-by-layer capsule was prepared and used for cellular uptake and MRI for human osteosarcoma, adenocarcinoma cells, and normal human mesenchymal stem cells.

Johnson et al. prepared theranostic nanoceria conjugated with fluorescein isothiocyanate-tagged epidermal growth factor receptor for the diagnosis and treatment of melanoma [116]. The theranostic potential of the nanoformulation was validated using both two-dimensional (2D) monolayer cultures and three-dimensional (3D) spheroid models derived from parental and metastatic melanoma cell lines. Confocal microscopy confirmed the diagnostic capability of the system through clear visualization of cellular uptake and distribution. To assess therapeutic efficacy, cell viability assays and ROS measurements were performed. In 2D models, a marked increase in overall cellular ROS was observed, while mitochondrial ROS remained largely unaffected. In contrast, the 3D melanoma spheroids exhibited a significant rise in both total and mitochondrial ROS levels, with metastatic spheroids showing a more pronounced response compared to the parental ones. These findings suggest that the nanoformulation is particularly effective against metastatic melanoma, highlighting its promise as a dual-function theranostic agent.

### 4.4. Nanoceria for Tissue Engineering Applications

Due to its strong antioxidant capabilities, nanoceria plays a crucial role in promoting tissue repair by aiding stem cell growth and guiding their transformation into specific cell types. It also encourages the formation of new blood vessels, thereby accelerating the healing of damaged tissues. When integrated into biomaterials and scaffold structures, nanoceria helps create environments that closely resemble natural tissue, which supports tissue regeneration and minimizes immune system rejection. Owing to its dual antioxidant and anti-inflammatory effects, nanoceria has emerged as a promising multifunctional tool for regenerating both soft and hard tissues, including skin, bone, nerve, and heart tissue [117–119].

Tissue repair facilitated by biomaterials generally follows two primary pathways: one involves regenerative remodeling, where damaged tissue is replaced with functionally similar parenchymal cells, and the other leads to the formation of fibrous tissue, resulting in scar-like structures. These outcomes are largely influenced by factors such as the regenerative capacity of resident cells and the severity of tissue damage, as well as the structural integrity or degradation of the extracellular matrix (ECM) at the implantation site [84,120]. A critical component in enhancing cellular regeneration lies in designing scaffolds that can replicate biochemical cues essential for parenchymal cell growth and ECM synthesis. Within the realm of regenerative medicine, stem cells serve as key agents for tissue reconstruction, often in combination with scaffolds that support their differentiation and integration [117]. The regenerative potential of these scaffolds often hinges on how well they interact with stem or progenitor cells. In this context, research has focused on exploring both the cellular microenvironment and the specific responses elicited by nanoceria, as detailed in Table 1.

**Figure 7.**
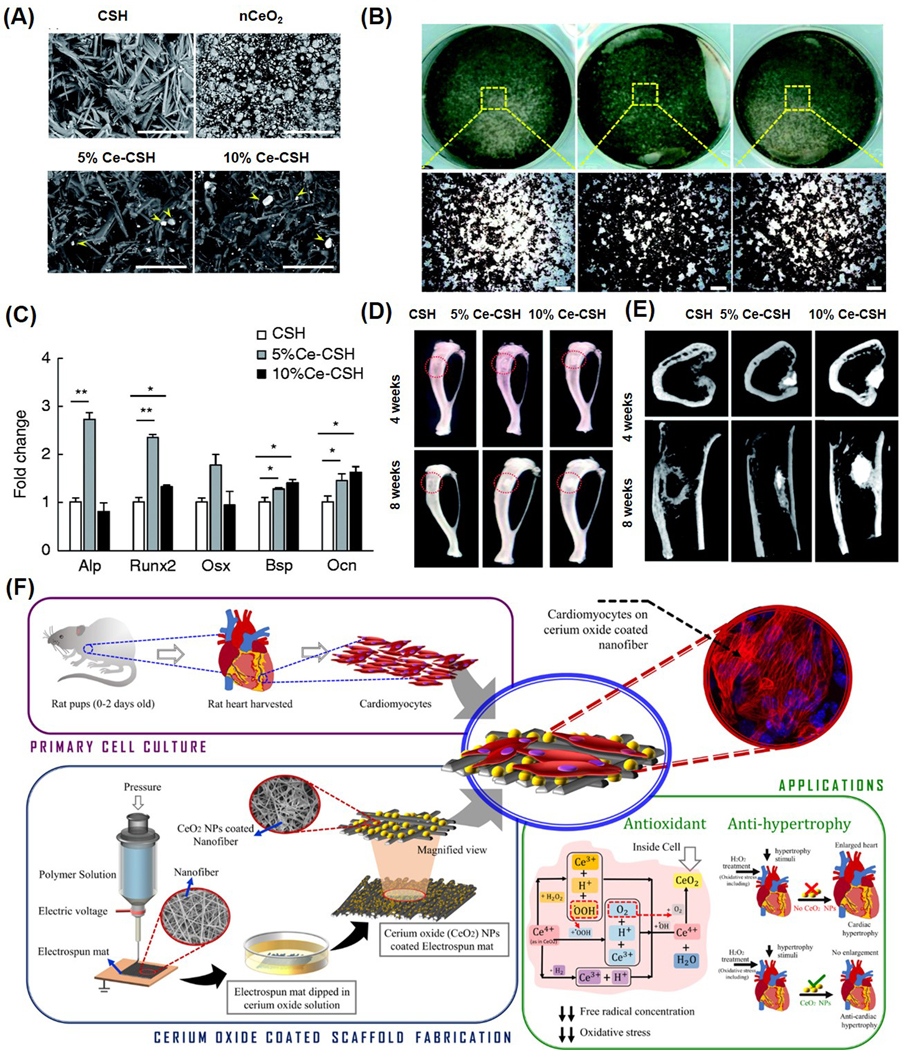
(**A**) Scanning electron micrographs for fabricated α-calcium sulfate hemihydrate (CHS)-CeO_2_ nanocomposite for bone tissue regeneration studies. Yellow arrows point to nCeO_2_ particles (**B**) von Kossa staining results showing mineral deposition in BMSCs following 21 days of treatment with different composite extracts. The scale bar in the lower panel represents 1 mm. **(C**) Quantitative analysis of mRNA levels for Alp, Runx2, Osx, Bsp, and Ocn in BMSCs after 7 days of incubation with extracts from various composite materials. Data represent triplicate samples per group (n = 3). *p < 0.05, **p < 0.01 vs. CSH group ( (**D**) X-ray images captured in the anteroposterior view at four and eight weeks following implantation, with the red circles indicating the site of the bone defect. (**E**) Micro-computed tomography (micro-CT) scans of the tibia at eight weeks after composite implantation. The top row displays cross-sectional (transverse) views, while the bottom row shows sagittal views of the tibial bone. (**F**) A schematic representation of the PCL-Gelatin-Ce nanofiber formation and its application for cardiovascular tissue engineering. Reproduced with permission from [123,130]. Copyright, 2019, Sage Publishers and 2021, Elsevier.

Numerous experimental studies have validated the therapeutic benefits of both pristine and surface-modified nanoceria in various biomedical applications. As a notable example, nanoceria doped with samarium and modified with polyethylene glycol (PEG) chains were shown to boost endothelial cell proliferation, activate key angiogenic signaling pathways such as p38 MAPK and HIF-1α, and stimulate blood vessel development in chick embryo models [121]. Furthermore, integrating nanoceria into polymer-based structures has emerged as a strategic approach for promoting wound closure and skin regeneration. Three-dimensional scaffolds embedded with nanoceria have demonstrated strong potential as biomimetic platforms for replacing injured dermal tissue [122,123]. The integration of nanoceria into α-calcium sulfate hemihydrate (α-CSH) at 5% and 10% concentrations was studied to develop a composite for bone repair (Figure 7A). Cell extracts from α-CSH and CeO_2_/α-CSH composites were tested in vitro, revealing that the 5% CeO_2_ composite notably enhanced cell proliferation, migration, and osteogenic gene expression in bone marrow stromal cells (Figure 7B,C). In vivo, critical bone defects in rats treated with the 5% CeO_2_ composite showed improved bone regeneration, as evidenced by imaging (X-ray and micro-CT), mineral deposition at the bone interface, and increased osteocalcin expression (Figure 7D,E). Overall, the 5% CeO_2_/α-CSH composite demonstrated superior osteogenic potential, suggesting its promise as a bone graft substitute [123].

Nanoceria has emerged as a promising candidate in treating cardiac conditions due to its antioxidant and anti-inflammatory actions. It shows potential in mitigating myocardial reperfusion injury by reducing elevated ROS levels and has demonstrated cardioprotective effects against oxidative damage. In CP-1 transgenic mice, intravenous delivery of 15 nmol nanoceria significantly curbed left ventricular dysfunction and dilation within two weeks [131]. Cardiac progenitor cells, essential for heart regeneration, require a controlled microenvironment for optimal growth. Nanoceria, due to its redox activity, can mitigate oxidative stress in cardiac progenitor cell cultures. Pagliari et al. found that exposure to nanoceria (5–50 μg/mL) for 24 h did not impair CPC function and offered protection from H_2_O_2_-induced toxicity for up to a week [126]. The interplay between microRNAs and ROS plays a critical role in ischemia–reperfusion injury. A novel composite of silica-polydopamine/DNA/nanoceria was developed by Yang et al. to test in cell and animal models. Their findings confirmed a regulatory interaction between H_2_O_2_ and miR-21 via the PI3K/AKT pathway, offering new insights into oxidative stress signaling [132]. Additionally, nanoceria-loaded electrospun polycaprolactam PCL and PCL-gelatin nanofiber patches were designed as antioxidant cardiac scaffolds (Figure 7E) [130]. These patches supported various heart cells and helped prevent hypertrophy in cardiomyocytes by neutralizing ROS.

### 4.5. Nanoceria for Wound Healing Applications

As the largest organ by surface area, the skin plays a critical role in safeguarding the body, making the rapid healing of topical wounds vital for restoring its protective function. Wound healing is a complex, staged process involving hemostasis, inflammation, proliferation, and remodeling [133]. During the inflammatory phase, various immune cells are recruited to secrete cytokines and chemokines that drive tissue repair. ROS, key signaling molecules in this phase, help eliminate pathogens and sustain the inflammatory response [134]. However, excessive or uncontrolled ROS production can prolong inflammation, leading to chronic, non-healing wounds. Nanoceria offers therapeutic potential by modulating ROS levels, thereby reducing oxidative stress and promoting the resolution of inflammation. Although they cannot penetrate intact skin, open wounds provide an ideal route for delivering nanoceria with high bioavailability [83,135].

Chigurupati et al. studied the use of bare nanoceria for wound healing applications [136]. Nanoceria, with an average size of 3–5 nm, were introduced to normal cutaneous wounds in mice. Nanoceria enhanced the in vitro proliferation and migration of keratinocytes, fibroblasts, and vascular endothelial cells. In vivo, topical application of these nanoparticles significantly reduced wound size in C57BL/6 mice compared to untreated controls. Histological analysis revealed an increased density of blood vessels and infiltration of mononuclear leukocytes, suggesting enhanced angiogenesis that may facilitate infection prevention and debris clearance: critical steps in the formation of new skin tissue. Additionally, the antioxidant properties of the nanoceria led to reduced lipid and protein oxidation at the wound site, as evidenced by lower levels of 4-hydroxynonenal and nitrotyrosine.

**Figure 8.**
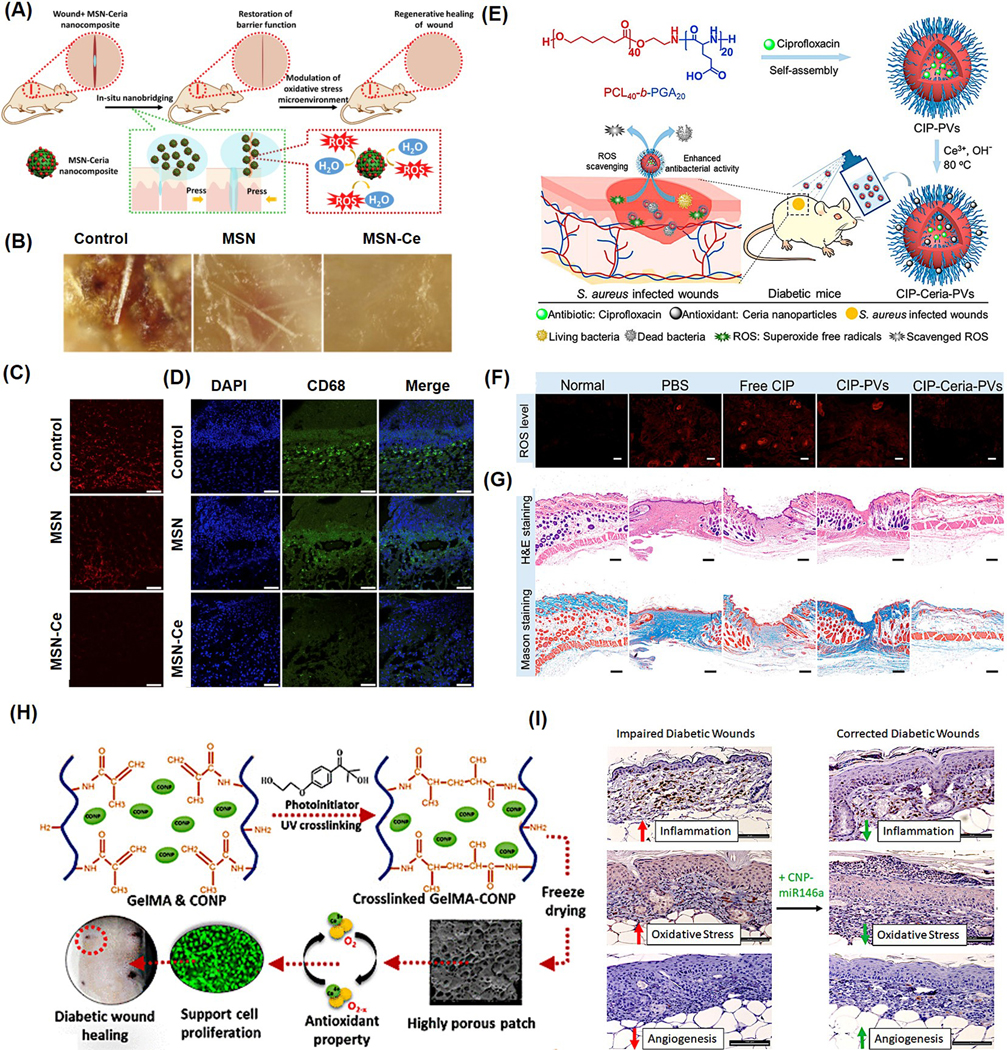
Application of nanoceria for wound healing applications. (**A**) Schematic showing accelerated wound healing using MSN-Ceria, a ROS-scavenging tissue adhesive that promotes wound closure by drawing wound edges together and mitigating oxidative stress to support tissue regeneration. (**B**) Stereomicroscopic images of wound surfaces on day 22 post-injury reveal healing progress. (**C**) DHE-stained cryosections on day 2 highlight superoxide anion production at the wound site. Scale bar: 100 μm. (**D**) Immunofluorescence images of CD68+ macrophages (green) on day 5 indicate immune cell infiltration in untreated, MSN-, and MSN-Ceria-treated groups. Scale bar: 50 μm. (**E**) Schematic illustration of the preparation and working principle of CIP-Ceria-PVs for diabetic wound application. (**F**) In vivo superoxide levels monitored by fluorescence microscopy using CIP-Ceria-PVs. Scale bar: 100 μm. (**G**) Hematoxylin and eosin-stained (H&E) or Masson-stained mouse skin tissue of the wound area. Scale bar: 200 μm. (**H**) A schematic showing the Gelma-Cerium NP hydrogel patches for wound healing applications. (**I**) H and E staining results for diabetic wounds from rats. mRNA-Cerium NPs showed reduced inflammation, oxidative stress, and enhanced angiogenesis after the treatment compared to control studies. Scale bar 50 μm. Reproduced with permission from [137–140]. Copyright 2018, Elsevier; 2021 and 2020, American Chemical Society; 2022, Elsevier, respectively.

For topical and diabetic wound closure, nanoceria was mixed with various adhesives or incorporated into various hydrogel patches. This allows the slow release of nanoceria at the wound site and reduces their toxicity. Wu et al. prepared 5 nm-sized nanoceria and incorporated it into mesoporous silica nanoparticles (MSNs) for topical wound healing applications (Figure 8A) [137]. The nanocomposite showed ROS scavenging properties, accelerated wound closure, and reduced scar formation (Figure 8B). A higher expression of messenger ribonucleic acid (mRNA) levels of stearoyl-CoA desaturase 1, leucine-rich repeats and immunoglobulin-like domain 1, placenta-expressed transcript-1, and platelet-derived growth factor-α in Sprague–Dawley (SD) rats were observed after the administration of NPs. The combined action of nanoceria, functioning as a catalytic antioxidant, and MSNs serving as tissue adhesives, was further validated by a noticeable decrease in superoxide anion levels and reduced infiltration of CD68-positive macrophages at the wound site (Figure 8C,D).

In diabetic wounds, elevated glucose levels lead to protein glycation, triggering the release of pro-inflammatory cytokines and disrupting healing by increasing oxidative stress and altering the extracellular matrix. These chronic wounds are prone to infection, often requiring antibiotics and immune modulators. Nanoceria, with their catalytic antioxidant properties, help reduce inflammation and oxidative stress, promoting healing in both standard and diabetic chronic wounds. Wang et al. fabricated polymeric vesicles (PVs) for the incorporation of antibiotic ciprofloxacin (CIP) and nanoceria for the treatment of infected wounds in a streptozocin (STZ)-induced diabetic mouse model (Figure 8E) [138]. The study was based on the understanding that diabetic individuals often have diminished levels of SOD, which controls oxidative stress. To address this, the researchers incorporated nanoceria, which possesses SOD-like properties, to promote more effective wound healing. To prepare the formulation, CIP-loaded PVs were incubated with cerium nitrate and sodium hydroxide, triggering the in situ formation of nanoceria on the vesicle surface. These nanoparticles, approximately 4 nm in size, were embedded into the PVs, resulting in a hydrodynamic diameter of 539 nm. The nanoparticles exhibited SOD-mimicking activity and facilitated the sustained release of CIP. Notably, the surface of the nanoceria contained 40.4% Ce^3+^, which can be an added advantage for antioxidant properties. Topical application of the CIP–ceria–PV system to wounds infected with Staphylococcus aureus in diabetic mice markedly accelerated wound closure (Figure 8F). This therapeutic effect was attributed to a dual action: bacterial eradication by CIP and reduction in ROS levels through antioxidant activity of nanoceria. The combined effect led to full wound healing and re-epithelialization within 14 days, whereas untreated and control groups continued to exhibit unhealed, open wounds without new epidermal formation (Figure 8G).

Various hydrogel-based scaffolds embedded with nanoceria are used for various wound-healing applications. The hydrogel polymeric support will allow the slow release of nanoceria at the wound site. Hydrogels provide a 3D network structure that can provide moisture, promote cell migration, and provide the ideal architecture for cell growth and tissue engineering, while also promoting the antioxidant properties of nanoceria [141,142]. Augustine et al. fabricated ceria-loaded gelatin methacryloyl (GelMA) scaffolds to enhance the healing of diabetic wounds [139] (Figure 8G). This composite material showed inhibition of bacterial growth and enhancement of angiogenic activity, leading to an acceleration in the healing process of diabetic skin wounds in rats with a loading efficiency of 1% w/w cerium in Gelma. Similarly, in two independent studies, it was reported that cryogels loaded with microRNA-nanoceria showed enhanced cell proliferation and wound healing capability in diabetic wounds [140,143]. Nanoceria exhibit strong antioxidant capabilities, while microRNA-146a serves as a suppressor of the NF-κB-mediated inflammatory response. When combined, the CNP-miR146a formulation offers a dual effect by simultaneously mitigating oxidative damage and inflammation. Administering this combination directly into the skin has been shown to improve collagen production, stimulate new blood vessel formation, and reduce inflammatory and oxidative markers, leading to accelerated healing in diabetic wound models (Figure 8H).

To better understand the multifaceted roles of nanoceria, numerous studies have been performed into their biological behavior and potential therapeutic benefits for wound healing. Pandey et al. observed that temperature significantly influences the redox functionality of CeO_2_ NPs, noting a decline in superoxide dismutase-like activity as temperature decreases [144]. Additionally, these nanoparticles demonstrated up to 60% inhibition of α-amylase at 1 mM, highlighting their relevance for antidiabetic applications, particularly in managing diabetic wounds due to their combined antioxidant, antimicrobial, and metabolic regulatory properties. In parallel, Bai et al. emphasized the enzyme-like capabilities of nanoceria, describing their redox mechanism involving the interconversion of Ce^3+^ and Ce^4+^ states, which creates oxygen vacancies [77]. This structural characteristic allows them to mimic multiple enzymatic functions, such as those of catalase, peroxidase, oxidase, and superoxide dismutase. These multienzyme-mimetic properties make nanoceria a promising tool in areas such as disease diagnostics, therapeutics, and broader biomedical applications, especially due to their capacity to neutralize nitric oxide and other reactive species. Further insights from He et al. explored the molecular underpinnings of nanoceria-mediated wound healing, identifying their role in modulating mitochondrial function via the NLRP3 inflammasome pathway [145]. Differential gene expression analysis pointed to involvement in immune regulation, metabolic pathways, and inflammatory signaling, including the TNFR2/NF-κB axis. Notably, CeO_2_–Y@ZIF-8@Gel treatment helped prevent mitochondrial DNA escape and reduced inflammasome activation, leading to decreased IL-1β secretion and promoting an anti-inflammatory macrophage phenotype by limiting cGAS-STING signaling.

Despite the potential of conventional nanozymes, their inability to continuously neutralize newly formed reactive oxygen species, especially hydroxyl radicals (·OH), poses limitations. Addressing this, Zhu et al. developed an advanced antioxidant nanoplatform by loading the NF-κB inhibitor JSH-23 into copper-doped ceria nanozymes [146]. To enhance tissue compatibility and adhesion in wound environments, these nanozymes were incorporated into a hydrogel spray composed of oxidized sodium alginate and methacrylated gelatin. This formulation not only activated Nrf2 pathways in macrophages, thereby suppressing oxidative stress at the source, but also maintained strong ROS-scavenging properties, significantly improving healing in diabetic wound models. Lastly, Carvajal et al. demonstrated that nanoceria can counteract oxidative stress-induced cellular changes by reversing H_2_O_2_-triggered phosphorylation events that regulate cell proliferation, stress signaling, and transcription [147].Their intervention notably impacted pathways involving mTOR, MAPK/ERK, CK2A1, and PKACA, further supporting the regulatory potential of nanoceria in oxidative stress-related cellular processes. The applications of nanoceria for wound healing are represented in Table 2.

Even though there are multiple examples and applications of nanoceria for wound healing, future research on nanoceria should focus on optimizing their formulation and delivery, understanding their molecular mechanisms, ensuring biosafety, and translating preclinical successes into clinical therapies using standardized protocols. Additionally, regulatory compliance should be considered. Caution must be exercised regarding potential toxicity, immune responses, and chronic effects of particles to fully realize their promise in wound care.

## 5. Biosafety and Toxicity of Nanoceria

The biosafety and toxicological evaluation of nanoceria is of paramount importance. Nanoceria’s unique redox capabilities mediated by the reversible cycling between Ce^3+^ and Ce^4+^ oxidation states confer antioxidant properties that are being explored for applications ranging from neuroprotection to drug delivery. However, despite its therapeutic promise, there remains considerable debate regarding its biological safety [41]. Experimental outcomes have been inconsistent, with some studies reporting cytoprotective and anti-inflammatory effects, while others highlight oxidative damage, genotoxicity, and organ-specific toxicity [148]. These discrepancies can be attributed to multiple variables, including synthesis route, particle size, morphology, surface chemistry, dose, exposure duration, and the choice of biological model. Nanoceria synthesized through eco-friendly routes, such as marine-mediated green synthesis, has demonstrated enhanced biocompatibility, exhibiting minimal toxicity in normal mammalian cell lines [149]. Surface modification strategies have further contributed to reducing toxicity; for instance, nanoceria coated with poly(acrylic acid) displays superior colloidal stability and reduced aggregation in physiological environments compared to citrate-stabilized analogs, resulting in diminished adverse cellular responses [150]. These findings highlight the critical role of surface engineering in improving nanoparticle compatibility with biological systems.

Nevertheless, the redox-active nature of nanoceria, while beneficial in scavenging ROS, can also act detrimentally under certain conditions [151,152]. In vitro and in vivo studies have shown that at elevated concentrations or with prolonged exposure, nanoceria may promote oxidative stress, leading to DNA damage, mitochondrial dysfunction, and inflammation [153]. For example, in rodent models, long-term oral administration of high doses (300–600 mg/kg/day) resulted in histopathological alterations in the liver, spleen, and brain, alongside indicators of genotoxicity and biochemical disruption [154]. Accumulation of nanoceria in major organs and its poor excretion rate may contribute to chronic toxicity, including granuloma formation and pulmonary fibrosis, particularly when larger or agglomerated particles are involved.

In vitro studies have also revealed complex interactions between nanoceria and various types of cells. Ultrafine particles (2–5 nm) exhibit enhanced cellular uptake and have demonstrated selective cytotoxicity toward cancer cell lines, such as human gastric carcinoma (BGC-803), while having minimal impact on healthy cells under controlled conditions [155]. Notably, surface functional groups influence intracellular uptake, oxidative stress levels, and cell viability. For instance, silanized nanoceria showed negligible cytotoxicity in healthy MRC-5 lung fibroblasts during short-term exposure, but extended exposure led to increased accumulation and toxicity [156]. Interestingly, human monocyte-derived macrophages exposed to nanoceria did not exhibit mitochondrial damage but instead displayed upregulation of anti-apoptotic proteins, suggesting a lower susceptibility to oxidative injury due to protective mitochondrial mechanisms and lower nanoparticle internalization [157].

Taken together, these findings underscore the dualistic nature of nanoceria. While it holds great potential as a therapeutic agent due to its antioxidant and catalytic functions, it also poses a risk of toxicity under specific physicochemical and biological conditions. To ensure its safe implementation in clinical and translational research, it is essential to establish standardized protocols for nanoparticle synthesis, surface functionalization, and toxicity assessment. Additionally, the Ce^3+^/Ce^4+^ ratio should be tightly regulated in nanoceria formulations, as it critically determines the redox reactivity and biological behavior of the nanoparticle. Future studies should focus on long-term biodistribution, biopersistence, and immune responses in both healthy and disease models to better define safe therapeutic windows for nanoceria. A general aspect of the advantages and disadvantages of nanoceria with other potential metallic nanoparticles for biomedical applications is depicted in Table 3.

## 6. Conclusions and Future Directions

The existing literature shows that nanoceria has emerged as a highly versatile nanomaterial with broad potential across diverse fields. Its distinctive redox and catalytic behavior has made nanoceria valuable in several biosensing and therapeutic applications. The excellent antioxidant properties of these nanoparticles were utilized as a diagnostic tool for detecting the COVID-19 virus and for targeting respiratory infections [158]. The potential use of nanoceria in medicine remains promising, particularly when considering strategies aimed at engineering their surface characteristics to enhance efficacy and biocompatibility. Notably, nanoceria demonstrates dual redox behavior, acting as either a reactive oxygen species (ROS) scavenger or an inducer, depending on the microenvironment. This duality is particularly advantageous in oncology, where nanoceria has shown selective toxicity towards cancer cells while shielding healthy tissues from chemotherapy-induced oxidative stress. Furthermore, nanoceria can be functionalized with various therapeutic and diagnostic agents, including small-molecule drugs, miRNAs, monoclonal antibodies, and imaging probes to enhance its biomedical performance. Some of these conjugate systems have already yielded encouraging results in preclinical models. Advancing the design of these surface-modified nanoceria platforms could offer significant breakthroughs in cancer therapy and beyond.

Despite the immense therapeutic potential of nanoceria, significant concerns exist regarding its biological safety. One major hurdle is the variability introduced by different synthetic methods, which can alter the physicochemical characteristics of nanoceria and thereby influence its biological activity and safety. This inconsistency complicates the prediction of the nanomaterial’s behavior in biological systems. Additionally, regulatory frameworks for nanomaterials remain underdeveloped, and concerns about nanoparticle purity persist, as contaminants can lead to unintended toxic effects.

Nanoceria has been known to have cytotoxic, genotoxic, and neurotoxic effects, particularly after prolonged exposure [159]. Extended contact with nanoceria may induce lung fibrosis and granulomas in both lung and liver tissues. Paradoxically, sustained exposure can also trigger inflammation and elevate oxidative stress in specific organs, including the brain, where neurotoxic effects persist even after cerium is no longer detectable [154]. These adverse outcomes highlight the delicate balance between the beneficial antioxidant roles nanoceria plays and its potential to cause harm within biological systems.

Another challenge with nanoceria in medicine is its rapid renal clearance, which is attributed to its nanoscale size [160]. While quick elimination is often beneficial in reducing toxicity, it limits the duration over which nanoceria can exert therapeutic effects, detracting from its efficacy in treating chronic conditions. Ironically, the very features that make nanoceria attractive—small size, robust ROS scavenging activity, and surface valence cycling—also contribute to its toxicological risks and clearance issues [161]. Thus, the unique physicochemical traits that enable its medicinal promise simultaneously underpin the difficulties faced in safely harnessing nanoceria for long-term biomedical use.

## Figures and Tables

**Table 1.** Application of various nanoceria formulations in tissue engineering scaffolds.

Nano Formulation	Role of Nanoceria	Cell Type	Tissue Target	Tissue Repair	Outcome	Ref.
Nanoceria-incorporated hydroxyapatite (HA) coatings	Additive to scaffold	Bone marrow stromal cells (BMSCs)	Bone	Constructive remodeling	Enhances cell viability and osteogenesis, restores antioxidant defenses and gene expression and inhibits apoptosis, osteoclastogenesis, and oxidative stress.	[124]
Cancellous bone containing poly-L-lactic acid and nanoceria	Additive to scaffold	Mesenchymal stem cells (MSCs)	Bone	Constructive remodeling	Improvement of cell proliferation; prevents apoptosis via calcium channel activation and HIF-1α stabilization.	[119]
Nanoceria	Dispersion in medium	BMSCs, bone and adipose	Bone	Constructive remodeling	BMSC viability increased, while osteogenic and adipogenic differentiation were inhibited in a time- and dose-dependent manner.	[125]
Nanoceria	Dispersion in medium	Cardiac progenitor cells (CPCs)	Heart	Constructive remodeling	No alteration of the cellular growth and differentiation; protection of cells against oxidative insults.	[126]
Citrate-stabilized nanoceria	Dispersion in medium	Primary mouse embryonic fibroblasts	-	Constructive remodeling	Enhanced proliferative activity of primary cells; reduction in intracellular ROS during the lag phase of cell growth; modulation of major antioxidant enzymes.	[127]
Nanoceria	Dispersion in medium	Human adipose derived-mesenchymal stem cells (hAd-MSCs)	Skin	Constructive remodeling	Improved tensile strength of acellular dermal matrices impregnated with nanoceria enhances hAd-MSC growth and survival, boosts free radical scavenging, and increases collagen content.	[128]
Nanoceria and samarium-doped nanoceria	Dispersion in medium	Neural progenitor cells	Nerves	Constructive remodeling	NPs enter cells and temporarily protect against oxidative stress. They hinder neuronal differentiation and disrupt the cytoskeleton, posing neurotoxicity risks. High collagen levels are observed.	[129]

**Table 2.** Applications of nanoceria for wound healing.

Nano Formulation	Scaffold/Wound Dressing Type	In Vitro	In Vivo	Outcome	Ref.
Nanoceria (3–5 nm)	Topical	Human keratinocyte cells	C57BL/6 mice	Enhanced cell proliferation and migration of keratinocytes, fibroblasts, and vascular endothelial cells; Reduced wound size in C57BL/6 mice; Increased density of blood vessels and infiltration of mononuclear leukocytes.	[136]
Nanoceria (5 nm) incorporated into mesoporous silica nanoparticles	Topical	HaCaT cells	Sprague–Dawley (SD) rats	Showed ROS scavenging properties; accelerated wound closure, and reduced scar formation; noticeable decrease in superoxide anion levels and reduced infiltration of CD68-positive macrophages at the wound site.	[137]
Nanoceria/poly(ε-caprolactone)-block-poly (glutamic acid)/Ciprofloxacin	Topical	Human normal liver cells	Streptozocin (STZ)-induced diabetic mouse model	Advantage for antioxidant properties; high antibacterial properties at wound site; full wound healing and re-epithelialization within 14 days.	[138]
Nanoceria-loaded gelatin methacryloyl (GelMA)	UV-crosslinked hydrogels	HaCaT keratinocytes and 3T3 fibroblasts	Diabetic male SD rats with full-thickness excision wounds	Highly porous structure; strong free radical scavenging properties; supports enhanced growth of 3T3 fibroblasts and HaCaT keratinocytes; facilitates re-epithelialization processes.	[139]
Nanoceria/miR146a	Intradermal injection	Murine non-diabetic or diabetic fibroblasts	Diabetic mouse model and a diabetic porcine model	Modulated both oxidative stress and inflammation; increases wound collagen, enhances angiogenesis, and lowers inflammation. Promoted faster closure of diabetic wounds.	[140]
Nanoceria NPs	In vitro studies	E. coli (gram + ve) and Enterobacter (gram − ve)	Not reported	Inhibited the alpha-amylase activity up to the 60%. Mitigated the oxidative stress-related disorder.	[144]
Nanoceria–Y@ZIF-8@Gel	Hydrogel	S. aureus and E. coli.	Diabetic wounds	High antibacterial, anti-inflammatory and wound adaptability; modulated the macrophage reprogramming-angiogenesis crosstalk to boost diabetic wound repair	[145]

**Table 3.** A comparison of the advantages and disadvantages of nanoceria with other metallic nanoparticles.

Feature	Nanoceria	Gold Nanoparticles (AuNPs)	Silver Nanoparticles (AgNPs)	Iron Nanoparticles (FeONPs)
Primary Use	• Biomedicine • Catalysis • Energy storage	• Biosensors • Bioimaging • Photothermal therapy • Targeted drug delivery • Cancer therapy	• Antimicrobial agents • Antiviral agents • Antifungal agents • Antineoplastic • Antioxidant • Antidiabetic	• Targeted drug delivery • Hyperthermia • Bioimaging • Cell labeling • Gene delivery • Environmental remediation
Advantages	• Antioxidant/multienzyme mimetic activity • Biocompatibility • Targeted action in cancer therapy • Enhanced stability with PEGylation	• Biocompatibility • Tunable optical/electronic properties • Facile surface functionalization • High stability • Non-cytotoxic to normal cells	• Broad-spectrum antimicrobial properties • Stable and less chemically reactive • High surface area	• Superparamagnetism • Chemical stability • Non-toxicity • Biocompatibility • High saturation magnetization
Drawbacks and Challenges	• Contradictory toxicity/activity reports • Challenges in biological utilization (protein corona) • Stability/agglomeration issues • Pro-oxidant effects • Variability based on preparation	• Toxicity concerns • Stability issues • Scalability challenges • Potential costs for treatment	• Toxicity (oral, immunotoxicity, neurotoxicity, environmental, reproductive) • Instability in biological environments • Can induce oxidative stress	• Instability under biological conditions (metallic iron) • Requires surface modification for enhanced stability/biocompatibility • Potential toxicity (dose-dependent)
